# Leveraging human resources for outbreak analysis: lessons from an international collaboration to support the sub-Saharan African COVID-19 response

**DOI:** 10.1186/s12889-022-13327-1

**Published:** 2022-05-31

**Authors:** Sara Botero-Mesa, Flavio Codeço Coelho, Kenechukwu Nwosu, Bertil Wicht, Akarsh Venkatasubramanian, Olena Wagner, Camille Valera, Benedict Nguimbis, Daniel Câmara, Izabel Reis, Lucas Bianchi, Morteza Mahdiani, Papy Ansobi Onsimbie, Papa Amadou Niang Diallo, Léa Jacques, Artur Manuel Muloliwa, Moussa Bougma, Leckson Mukavhi, Adit Kaneria, Ram Peruvemba, Ajay Gupta, Isotta Triulzi, Ananthu James, Verena Carrara, Wingston Ngambi, Zahra Habibi, Michael Tedros Adhanom, Sabina Rodriguez Velásquez, Paolo Sestito, Timokleia Kousil, Loza Biru, Daniela Vivacqua, Jyoti Dalal, Anatole Mian, Maroussia Roelens, Erol Orel, Cristina Barroso Hofer, Fatihiyya Wangara, Franck Mboussou, Tamayi Mlanda, Arish Bukhari, Theresa Min-Hyung Lee, Roland Ngom, Beat Stoll, Cleophas Chimbetete, Jessica Abbate, Benido Impouma, Olivia Keiser

**Affiliations:** 1 Institute of Global Health, Faculty of Medicine, University of Geneva, Geneva, Switzerland; 2 The Global Research and Analysis for Public Health (GRAPH) Network, Association Actions en Santé, Geneve, Switzerland; 3 School of Applied Mathematics, Getulio Vargas Foundation, Rio de Janeiro, Brazil; 4 Faculté de Lettres, University of Lausanne, Lausanne, Switzerland; 5 Institute for Technology and Global Health, Massachusetts Institute of Technology’, Cambridge, USA; 6 Transform Health Coalition, Geneva, Switzerland; 7 Data analysis, The GRAPH Network, Douala, Cameroon; 8 Laboratório de Mosquitos Transmissores de Hematozoários, Instituto Oswaldo Cruz, Fundação Oswaldo Cruz, Brasil - LATHEMA/IOC/FIOCRUZ, Rio de Janeiro, Brazil; 9 Núcleo Operacional Sentinela de Mosquitos Vetores, Fundação Oswaldo Cruz, Brasil - NOSMOVE/FIOCRUZ, Rio de Janeiro, Brazil; 10 Sergio Arouca National School of Public Health, Fundação Oswaldo Cruz, Brasil - ENSP/FIOCRUZ, Rio de Janeiro, Brazil; 11 Research and Training Unit in Ecology and Control of Infectious Diseases (URF-ECMI), Faculty of Medicine, University of Kinshasa, Kinshasa, Congo; 12 National Aids Committe, Fann Hospital Center, Dakar, Senegal; 13 Faculty of Medicine, University of Geneva, Geneva, Switzerland; 14 Faculdade de Ciências da Saúde, Universidade Lúrio, Nampula, Moçambique; 15 Institut Supérieur des Sciences de la Population (ISSP), Université Joseph KI-ZERBO, Ouagadougou, Burkina Faso; 16 Faculty of Medicine and Health Sciences, University of Zimbabwe, Harare, Zimbabwe; 17 School of Information Studies, Syracuse University, Syracuse, NY USA; 18 HSR.health, Rockville, MD USA; 19 Institute of Management, Scuola Superiore Sant’Anna, Pisa, Italy; 20 Department of Chemical Engineering, Indian Institute of Science, Bangalore, India; 21 Centre for Tropical Medicine and Global Health, Nuffield Department of Medicine, University of Oxford, Oxford, UK; 22 Health Economics Policy Unit, Department of Health Systems and Policy, Kamuzu University of Health Sciences, Lilongwe, Malawi; 23 Pediatric Infectious Diseases, Federal University of São Paulo, São Paulo, Brazil; 24 Data analysis, The GRAPH Network, Abidjan, Ivory Coast; 25 Department of Infectious Diseases, Universidade Federal do Rio de Janeiro, Rio de Janeiro, Brazil; 26 Department of Health Services, County Government of Kwale, Kwale, Kenya; 27 World Health Organization, Regional Office for Africa, Brazzaville, Congo; 28 Association Actions en Santé Publique, Geneva, Switzerland; 29 Newlands Clinic, Harare, Zimbabwe; 30 UMI TransVIHMI (Institut de Recherche pour le Développement, Institut National de la Santé et de la Recherche Médicale, Université de Montpellier), Montpellier, France; 31 Geomatys, Montpellier, France

**Keywords:** Sub-Saharan Africa, COVID-19, Outbreak, Pandemic, Health emergency, Data management

## Abstract

Emerging infectious diseases are a growing threat in sub-Saharan African countries, but the human and technical capacity to quickly respond to outbreaks remains limited. Here, we describe the experience and lessons learned from a joint project with the WHO Regional Office for Africa (WHO AFRO) to support the sub-Saharan African COVID-19 response.

In June 2020, WHO AFRO contracted a number of consultants to reinforce the COVID-19 response in member states by providing actionable epidemiological analysis. Given the urgency of the situation and the magnitude of work required, we recruited a worldwide network of field experts, academics and students in the areas of public health, data science and social science to support the effort. Most analyses were performed on a merged line list of COVID-19 cases using a reverse engineering model (line listing built using data extracted from national situation reports shared by countries with the Regional Office for Africa as per the IHR (2005) obligations). The data analysis platform The Renku Project (https://renkulab.io) provided secure data storage and permitted collaborative coding.

Over a period of 6 months, 63 contributors from 32 nations (including 17 African countries) participated in the project. A total of 45 in-depth country-specific epidemiological reports and data quality reports were prepared for 28 countries. Spatial transmission and mortality risk indices were developed for 23 countries. Text and video-based training modules were developed to integrate and mentor new members. The team also began to develop EpiGraph Hub, a web application that automates the generation of reports similar to those we created, and includes more advanced data analyses features (e.g. mathematical models, geospatial analyses) to deliver real-time, actionable results to decision-makers.

Within a short period, we implemented a global collaborative approach to health data management and analyses to advance national responses to health emergencies and outbreaks. The interdisciplinary team, the hands-on training and mentoring, and the participation of local researchers were key to the success of this initiative.

**Supplementary Information:**

The online version contains supplementary material available at 10.1186/s12889-022-13327-1.

## Background

The WHO African region, which is made of 47 countries, is plagued by numerous infectious diseases. Each year, over 100 acute public health events are reported by Member States to the WHO Regional Office for Africa in line with the International Health Regulations (IHR 2005) [1]. Many of these emergencies result in high morbidity, mortality and socioeconomic disruptions, which threaten national, and regional health security. Infectious diseases account for 80% of these emergencies, with cholera, measles, and meningitis being the most common recurring outbreaks [2]. These outbreaks showed the need for robust and coordinated global infectious disease control regimes long before the pandemic [3, 4]. However, the unique characteristics of COVID-19, the first disease which affected all 47 member states simultaneously since the adoption of the WHO Emergency Response Framework [5] highlighted the critical role of WHO in overseeing and coordinating the regional response as well as the limited availability of human resources to support it [6, 7].

Half the world lacks access to essential health services, and there is a global shortage of health workers [8, 9]. Africa has 12.8 skilled health professionals per 10,000 population compared with the Americas and Europe, which have 82.6 and 105.3 per 10,000 population, respectively [10]. Limited capacity and funding are key problems facing researchers in Africa [11]. The continent has less than 0.5% of world R&D expenditures and even fewer students who opt for research careers [12]. The 7th African Regional Conference of Vice Chancellors and Deans of Science, Education, Engineering and Technology (COVIDSET), accentuated the startling statistics of how few African researchers are involved in international projects and collaboration. During the conference, delegates raised concern about how limited their collaboration were with other countries on the continent and beyond. This means a lot of research conducted in Africa does not feed into global research efforts.

The ongoing response to the pandemic in the WHO African region has created an unprecedented data demand and use from member states, partners and other stakeholders. To address this challenge, the Regional Office for Africa through its WHO Health Emergencies Programme, set-up an information management strategy and issued a call for expertise in COVID-19 data analytics. The purpose of this paper is to describe the experience and the lessons learned from a joint project with WHO AFRO to support the sub-Saharan African COVID-19 response. The paper also highlights solutions around developing and implementing a global collaborative approach to health data management for epidemiological analysis during health emergencies and outbreaks.

## Main text

### Developmental process and resources

#### The global research and analyses for public health (GRAPH) network

The COVID-19 pandemic situation in the African Region was characterized by an exponential increase in the number of cases and deaths affecting all Member States concurrently. The sheer scale and magnitude of the pandemic in the region led to the urgent need to expand HIM-WHO AFRO (Health Emergency Information and Risk Assessment - World Health Organization Africa) efforts in critical areas such as data infrastructure or platforms, epidemic intelligence, high-powered data analytics, documentation, automation and visualization, and reporting to meet the pace of the pandemic and the demands from Member States [7].

In June of 2020 the HIM-WHO AFRO mandated Association Actions en Santé Publique (ASP) and its partner the division of Infectious Diseases and Mathematical Modelling of the University of Geneva (UNIGE) to support their efforts in these areas of work.

Considering the complexity of the situation, and the wide scope of work, ASP and UNIGE decided to open a call within their networks of experts and collaborators for supporting WHO AFRO’s COVID-19 response efforts. A wide range of academic centers and international health organizations responded to the call, giving rise to a multinational team of global health researchers that led to the creation of a dedicated network, called The Global Research and Analyses for Public Health (GRAPH) Network [13]. African data analysts were contacted and integrated into the team thanks to professional contacts of GRAPH Network members.

**Fig. 1:**
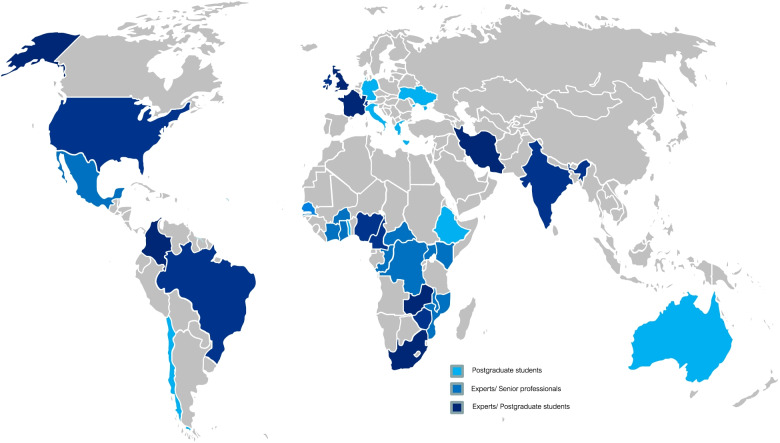
Residence countries of members of the GRAPH Network. Legend: The multidisciplinary research network consists of 63 collaborators in 32 countries. Source: “File:BlankMap-World.svg.” Wikimedia Commons, the free media repository. 15 Jul 2021, 00:12 UTC. 27 Jul 2021, 12:51 https://commons.wikimedia.org/w/index.php?title=File:BlankMap-World.svg&oldid=575140996

The Network successfully mobilized 63 professionals and postgraduate students of 32 nationalities including 17 sub-Saharan African countries, 7 European, and 5 North and South American countries plus, India, Iran and Australia (Fig. 1). Ten disciplines were represented, ranging from social science, public health and epidemiology, to data and computer science.

#### Teams and workflow

The GRAPH Network supported young professionals to build their career path as global health data analysts and outbreak response professionals. Individuals currently employed elsewhere or in retirement volunteered as experts, while students and data analysis experts from African countries and other countries outside Africa were given a small financial compensation for their work.

The group was organized into three main sub-teams led by a coordination team. These were the reporting team, the data analysis team and the geospatial team. The reporting team was responsible for literature search, data extraction and interpretation, and report writing and editing. The data analysis team focused on creating the R Markdown scripts to process the data in an automated way and developing the script templates for producing the epidemiological indicators from the cleaned data. The geospatial team worked on risk mapping, with the development of subnational mortality and transmission risk indices. Detailed description of each team is summarized in Table 1.

**Table 1:** GRAPH Network’s teams

Team	Description
**Reporting**	15 postgraduate students, 3 universities, 15 nationalities **Tasks:** literature search, data extraction and interpretation, and report writing and editing. **Supervision:** Team supervised by senior epidemiologists from Zimbabwe, Senegal, Brazil, Switzerland and France. **Tools:** Zoom, Cuicloud, weekly meetings.
**Data analysis**	18 postgraduate students from 9 universities, 30 data scientists and programming specialists, 24 nationalities. **Tasks:** This team divided into two sub-teams worked with primary data received from the field and created the R Markdown scripts to process the data in an automated way, yet tailored to each country. Programmers developed the script templates for producing the epidemiological indicators from the cleaned data while junior analysts cleaned the primary data and ran the scripts modified for each country. **Supervision:** The team was supervised by three senior data analysts and computer scientists from universities and research institutions in Montpellier, Geneva and Rio de Janeiro. **Tools:** Renku, Gitlab Rstudio, Rmarkdown, Slack, weekly meetings.
**Geospatial analysis**	The team of five experts from Uganda and a US-based partner institution worked on mortality and transmission risk index calculation.
**Coordination**	The association Actions en Santé Publique and the head of the division of Infectious Diseases and Mathematical Modelling of the University of Geneva provided the general guidance, political representation and ultimate decision making. The coordination was supported by a project manager and three senior analysts.

#### Data management

The network’s cloud infrastructure was based on The Renku Project (https://renkulab.io) [14], an integrated platform for collaborative data science. The Renku Project provided the data analysis teams with standardized and versioned containers for cloud computing which helped maintain a homogenous software environment (Renku) across all analysts. It also provided version control through an integrated instance of Gitlab [15]. Using Renku allowed for a homogeneous computation infrastructure to the network of analysts around the globe, only requiring access to an internet connection.

The team handled anonymized individual-level data and aggregated data shared by 28 member states with WHO/AFRO in accordance with the International Health Regulations and signed agreement between WHO/AFRO and ASP. The linelist data was uploaded to Renku where the whole data analysis team ran standardized scripts for data cleaning and data analysis. Network analysts also used other data sources such as the WHO’s own web-scraped COVID-19 dashboard data, ECDC (European Centre for Disease Prevention and Control), and open data organizations such as GADM [16] (for maps of the world’s administrative regions) and Gridded Population of the World [17] (for demographic data) among others. Analysts worked with data as flat files (CSVs, Shapefiles, etc.) stored on an internal Gitlab repository and versioned through Git-LFS [18].

**Fig. 2:**
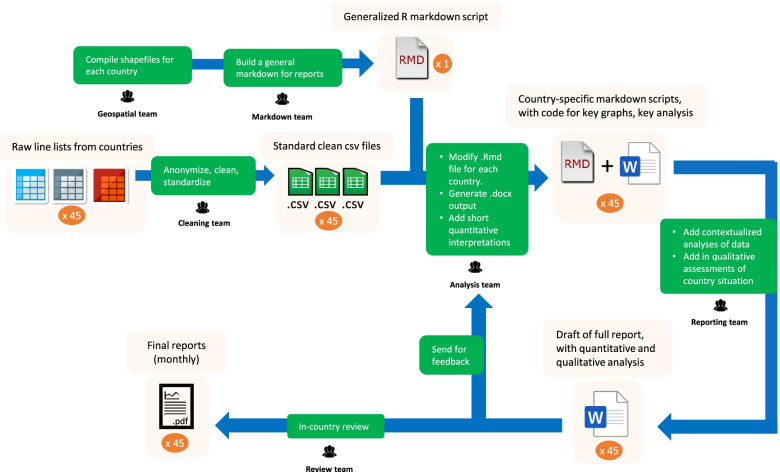
visual outline of the network’s workflow. Legend**:** The analysts’ output was provided in an Rmd-PDF, with the analysis code and high-resolution figures saved in a separate folder. This process was adapted and standardized for each country and was designed to be automated as much as possible (although some adaptations were needed for every new linelist as the formats changed frequently). Reporters (qualitative analysts) contextualized epidemiological data based on country-specific COVID-19 status whilst utilizing a standardized template

Through version control and code review workflows, the team produced standardized code and scripts enabling the reproducibility of monthly and weekly in-depth country specific reports. The main goal of this process was to generate efficient and well documented code easily used by novice analysts. Throughout the workflow (Fig. 2), automation was central as the team strived to maximize its throughput in the generation of the reports. Code reuse guidelines were also key to minimize coding errors and the duplication of efforts by the analysts.

#### Data analysis

The data analysis pipeline was under intense in-house development by the data analyst team. The code was written in R or Python and was versioned on git repositories hosted on the network’s Gitlab server (integrated with the Renku platform). The data analysis tasks were divided into three general categories:

- **Data-cleaning and harmonization:**
  The data-cleaning procedure was based on a code-book developed by the data-cleaning team through analysis of the raw data received from multiple countries. During this procedure the data was normalized, converted to achieve type homogeneity, and also harmonized with external datasets (e.g. DHS, DHIS2) to allow for linkage.
- **Epidemiological data analysis and statistical modeling:**
  The analysis code developed in the R language was used for calculating standard epidemiological indicators such as case incidence, attack rate, case fatality ratio, test positivity rate, information on contact tracing, etc. Data were presented in reports in the form of tables and figures. The visualization of the various epidemiological analyses was implemented in such a way to automatically guarantee optimal visualization of the information independently of the scale of the data. This was important since some countries reported only a few cases while others had tens of thousands of new cases over the same time period.

##### Reporting

The automated report generation pipeline was based on RMarkdown templates that used the analytical code to generate the reports according to a set of parameters specified. This design allowed for a large number of reports to be generated automatically from a few master templates. The RMarkdown template was compiled into PDF or Word documents that were passed on to the reporting team and epidemiologists who then conducted in-depth literature reviews using MOH (Minister of Health) situational reports and accredited news outlets to contextualize the analytical results with a qualitative discussion. The report templates were developed jointly with WHO AFRO, who facilitated direct communication with countries. Thus, our team was able to hold meetings with countries specifically to solicit and integrate any feedback they provided. Even though we had collaborators who worked closely with the minister of health in many countries, our communication with country authorities was mediated by WHO Afro. The creation of the templates and the content of reports was discussed with WHO Afro who had previously agreed with countries about the points of surveillance, the reports issued for our network were closely aligned to those discussions and parameters. WHO Afro transmitted the reports to the WHO national offices and to the Ministers of Health, following their review and approval.

The in-depth epidemiological reports were the main communication outputs from The GRAPH Network’s mandate from WHO AFRO for support with COVID-19 analytics. However, the dissemination and/or publications of outputs were determined exclusively by countries.

One example of the in-depth epidemiological reports developed by the network is provided in Additional file 1. COVID-19 Epidemiological Report. Mauritius. Example In-depth epidemiological report. One out of 45 developed by the GRAPH network.

#### Project evaluation: web survey

During the final stages of the collaborative project with WHO AFRO, postgraduate students, in-country experts, and other global health professionals were given the opportunity to reflect on their experience and views on the network through an anonymous online survey. Similar questions were asked to each group, with slight variations depending on team competency. Of the 63 members comprising the network, 51 individuals submitted the survey. The questionnaire is provided in Additional file 2. Web survey. Surveys were answered by postgraduate students, in-country experts, and other global health professionals.

### Outcomes

#### Network track record

- **In-depth epidemiological reports, rapid country updates and country profiles**

The network’s collaboration with the HIM-WHO AFRO resulted in the production of 45 in-depth country-specific epidemiological reports for 28 countries, namely, Niger, Namibia, Burkina Faso, Chad, Republic of Congo, Mauritius, DRC, Sao Tome Principe, Seychelles, Sierra Leone, Tanzania, Eswatini, Botswana, Ghana, Liberia, Cabo Verde, Equatorial Guinea, Guinea, Angola, Ivory Coast, Rwanda, Uganda, Senegal, Gambia, Kenya, Mozambique, Burundi, Zimbabwe. Weekly updated reports providing timely data updates were also developed for countries. These reports were used by the Ministries of Health and WHO national offices to guide decisions on the COVID-19 response.

In addition to the weekly and In-depth epidemiological rreports, the team also produced 32 country profiles and data quality reports on the data received from WHO member states. The country profiles and data quality reports (described below in detail) were useful to WHO AFRO to encourage countries to improve their data collection, management, and data sharing policies.

b) **Transmission and mortality risk index**

Transmission Risk Index and Mortality Risk Index maps were developed for the 23 countries. The maps were produced at the Admin 1 level and Admin 2 level (i.e. Province and District, depending on the administrative boundaries determined by each country) where the case data were available. The Transmission Risk Index identified the risk for potential spread and transmission of COVID-19. The Mortality Risk Index identified the regions with the highest risk of critical illness and death due to the virus. These were produced along with brief descriptions and interpretation of the maps in terms of COVID-19 response.

The Transmission and Mortality Risk indices were individually normalized for each country as well as across all 23 countries. When normalized for each country, the indices highlighted local level view of risk within each country, enabling in-country resource allocation and strategic implementation of mitigations (e.g., lockdowns). When normalized across all countries, the indices provided a view of relative risk among countries across the continent supporting resource allocation on a broader scale. Transmission Risk Index and Mortality Risk Index maps for the continent were also developed to provide a view of the risk for the continent.

c) **Building data analysis capacities**

African specialists became fundamental elements for the assessment of the in-depth epidemiological reports carried out by the network; their collaboration also made it possible to establish direct connections with ministries of health for the joint planning of projects to strengthen national epidemiological surveillance systems.

**Training**

The network brought together 34 postgraduate students from 11 different universities in Africa, Europe, North America, and Latin America. It provided a “real life” training environment for young professionals and students as they were exposed to applied data-science, interpretation, and presentation on a global scale.

Training started with a mentorship program that then turned into a training program for the new members, junior analysts, and local African analysts for performing data analysis according to the group’s pipeline. The training was addressed to analysts familiar with R and designed mainly to provide the essential tools and guidance to local analysts on the ground.

Newcomers benefited from peer mentorship and group leaders as they joined the network, and subsequently became mentors of new trainees themselves.

Currently, the GRAPH Network is developing online training modules for public health personnel in partner countries with plans to publish these courses on a platform, https://thegraphnetwork.training [19]. The training modules will serve to (a) enhance in-country expertise in the programmatic analysis of outbreak data, and (b) capacitate in-country partners to autonomously customize and manage their instance of the EpiGraphHub application.

**Data quality reports**

Through data-quality reports on the data shared by countries, analysts provided feedback on improvements to optimize COVID-19 data management and data standardization.

d) **EpiGraph hub**

WHO-AFRO developed the DSV Tool COVID-19 (DSV: Data Summarization and Visualization tool) at the beginning of the pandemic. The DSV tool consists of modules for data collection, automated data management and analytics, and visualization [20]. Aligned to this initiative the EpiGRAPH hub developed by our team would enhance two of the DSV modules a) automation of advanced data analysis and b) one-click visualization and reporting.

**Fig. 3:**
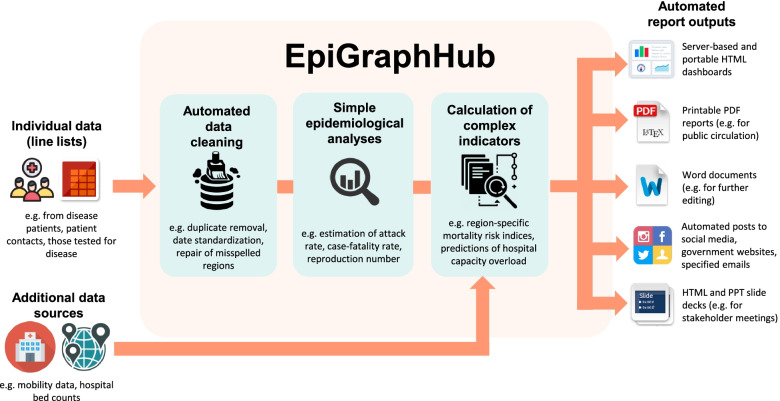
The EpigraphHub workflow. Legend: The EpiGraphHub application automates the production of epidemiological reports in multiple modalities

EpiGraphHub is a software platform designed and implemented by The GRAPH Network to incorporate all the work previously done and the technology developed by team members. The overall goal of this tool is the automation of the processes to generate the epidemiological situation and analysis reports. Through an interactive web-based dashboard with an intuitive user interface (UI), the processed information would be clear and actionable for decision makers who may not have a statistical background. Figure 3 Shows the EpigraphHub workflow.

The tool is much more than a dashboard. It is a living data analysis and visualization hub. Going beyond descriptive statistics, the hub aims to add advanced functionality (e.g. dynamic visualizations, decision scenario modeling, geospatial processing, derived risk indices) and crisis-ready responsiveness to the toolbox of logistics planning and analysis tools that are typically available to decision-makers. Moreover, the hub provides a common platform supporting data standardization practices that can allow for regional comparisons if and when data are shared. Operation and development of the Hub implicates strengthening the capacity of those closest to the ground for data stewardship and valorization.

The EpiGRAPH Hub’s capacity for data management and analysis is currently built for responding to emerging epidemics, but its versatility enables it to be used for surveillance, response, and assessment of control strategies for other endemic and epidemic diseases. Due to its adaptability, collaborative development structure, and ambitions for a range of future functionalities, there is no limit to the types of data that can be analysed or methods employed. A detailed description of the EpiGRAPH Hub is presented in Additional file 3. EpiGRAPH Hub Concept sheet.

#### Project evaluation: web survey

Overall consensus on the working experience was positive, with many highlighting the enriching interactions that occurred during the project. Postgraduate students and junior data analysts both emphasized the value of learning from highly skilled professionals (e.g., “*Everyday was a positive experience – such as learning about on the ground realities from a field operative, or collaborating on risk mapping with a geospatial expert”*, “*At the GRAPH, I was exposed to worldwide expertise on data analysis it is a wonderful learning experience*”). In-country experts also voiced similar views, commenting on the substantial benefits of a worldwide collaborative network (e.g., *“This experience as a GRAPH Network collaborator was very enriching, due to the fact that I broadened my field of competence as an epidemiologist, to integrate a network of researchers working One Health approaches. It allowed me to strengthen my capacities in the field of epidemiological surveillance, data management, and report writing”*).

With regard to capacity building and relevance of the network, all groups responded favorably once again. Involvement with the project not only gave students real-time experience in public health decision making during critical points of the pandemic, but also allowed for the application of various global health concepts through practical work (e.g., *“Writing reports that involve the different aspects that any global health expert should accomplish. Including being able to make analyses of socio-political situations and their relation with the global health issue in study and epidemiological responses*”). On the other hand, data analysts stated that they benefited from learning new tools such as Renku and junior analysts reportedly refined multiple areas of their skills whilst working with imperfect data (e.g., *“I am much more proficient in data cleaning and data analysis as well as version control, and collaborating on work”*). Professional experts also gained value from the experience through collaborative work with other experts on the team (e.g., *“The impact was very significant in the sense of improved knowledge on data processing. Personally, I have reinvested in deepening my knowledge of R*”, “*Being able to take part in high level discussion regarding data analysis and epidemiological analysis in a truly multicentric scope, and the dedication of the group to produce scientific publications”*).

Some suggested areas of improvement include ensuring a baseline access to technology for all members of the network since internet connectivity was one of the recurring issues faced. Alternatively, others suggested a standardization of the teaching and learning process for data cleaning as well as data analysis (e.g.*, “More coherent training materials for students”, “Same and consistent training for everyone”*). Nevertheless, all survey respondents stated that they would recommend the GRAPH network to support epidemiological responses in different contexts and organizations. Most notably, in-country experts expressed that they would recommend the network to support the Ministry of Health response within their own countries (e.g., *“To provide the country with the expertise to improve the availability of strategic information as soon as possible for decision making aimed at controlling epidemics”*, *“Besides having the technical capacity, GRAPH network has in-country collaborators across African countries. The combination of technical expertise and an in-depth understanding of the African context is key”*). The majority of comments received highlight the network’s international team, diverse expertise, and capacity building as its greatest assets for epidemiological response in various situations (e.g., *“The international dimension allows the GRAPH network to adapt quickly for different situations, and because the numerous skills present within the team can be ‘spread’ to all members through a very efficient structure*”).

#### Lessons learned

The main mandate of the GRAPH network // HIM-WHO AFRO collaboration project centered on data management, from which the idea of creating and promoting the implementation of EpiGRAPH, a tool that facilitates data management for outbreak response originated. Thus, the lessons learned presented here are based on the analysis of the challenges we encountered in both data management (Table 2) and data governance (Table 3) which is a cross-cutting and overarching aspect of data utilization in global collaboration platforms [21].

**Table 2:** Lessons learned by the GRAPH network on data management

Data management dimension	Lessons Learned.
**Data Collection**	− Quality control at the data cleaning step is primordial to preventing downstream problems. − Quantitative evaluation and constructive feedback to data providers improves data quality.
**Data Storage**	− Data files should systematically be archived, and file names standardized, searchable, and dated. − Analysts need to be trained to foresee data infrastructure issues, such as how to avoid problems with large data file formats. − Clear separation between original data, cleaned data and analytical results is key for reproducibility.
**Data Use**	− Data utilisation approaches must involve and be agreed to by the data provider − Studies must be designed with clear reasoning for how it serves the community from which the data originate.
**Results Dissemination**	− Dissemination of data and/or results of analyses requires large levels of mutual trust and meaningful collaboration between the Network and the data providers.
**Data Analysis**	− Data analysis and interpretation must be tailored to the context of each country or region’s specific situation. − Source code versioning and review are key tools for the development of correct and well documented code.

**Table 3:** Lessons learned by the GRAPH network on data governance

− Effective, equitable and participatory health is established through strong bonds of trust with partner countries. − Countries must be encouraged to ensure individuals are owners of their own health data. Health data needs to be a global public good, and its use in the public sphere should always consider the key role of national public health institutions. − Safety and security of data storage platforms must be prioritised to ensure respectful protection of confidential health data. − Crucial gaps around data ownership must be clarified to democratise health data: individuals must own their health data that they contribute. − The implementation of epidemiological surveillance data management tools should actively involve partner countries in all stages of the process to ensure sustainability over time, and data management autonomy.	

### Relevance to the field

We successfully implemented a global collaborative network that supported health data management and analysis during the COVID-19 health emergency in the WHO African region. Our experience ended up leveraging a bottom-up approach to build data science capacities at the local level.

The GRAPH network was established after several countries were affected by COVID-19. This meant that there was urgency to work on data management solutions since the need emerged during the outbreak response. The GRAPH network // HIM-WHO AFRO collaboration project has guided our thinking on how to address the problem of data management in Africa, especially during an outbreak. This approach offers a foundation for integrating data management into national contingency plans, with a view to moulding them into an efficient and effective instrument for regional and global transmission control (See Additional file 3. EpiGRAPH Hub Concept sheet.).

The main challenges that Africa faces in the response to life-threatening outbreaks pertain to limited local biotechnological production, limited access to the basic infrastructure needed for data acquisition and analysis, and limited research capacity or expertise in specialty fields [22–24]. The GRAPH network initiative tackled three of those challenges, leveraging human resources for outbreak analysis, strengthening outbreak response in African countries with programmatic tools for analysis and reporting and working as a bridge between countries, and international health organizations.

In 1990 the Commission on Health Research for Development recognized that health research is an essential part of the health system and that it plays a critical role in improving health outcomes [25]. Since then many programs ranging from capacity building to specific disease research in low- and middle-income countries have been launched and supported by a variety of international institutions. Although there have been many programs over the years to help build research capacity just a few countries in Africa have developed significant local research capacity capable of functioning on its own and ready to fully partner with international colleagues [22–24]. The human capacity for outbreak response in Africa is greater than numbers imply, but there are bottlenecks in translating that potential into action. Programs with top-down approaches normally limit the participation of local researchers and, with difficulty, manage to be integrated as a national program minimizing in this way the possibility of being sustained over time. The problems inherent to “parachute science” have been well documented, and people in the field advocate for overseas researchers to collaborate with local scientists [26]. There is a growing call for bottom-up approaches to research that enhance local capacity so that it stays and reproduces there [27]. The strength of the GRAPH network and what sets it apart from many other initiatives is its bottom-up approach.

In general, programs and projects that seek to support capacity building have focused on the training of individual researchers, leaving aside the integration of national institutions that participate in the generation and dissemination of health knowledge. The number of trained researchers is essential but a national health research system goes far beyond it and must include local institutions and activities at all levels of research production [24]. The GRAPH network looked actively for specialists integrated in the academic sector that were close advisers of MoH. The rationale behind this strategy was to reinforce human capacity and cooperation between academia and governmental agencies to facilitate the implementation of long term strategies for epidemiological surveillance and outbreak response that are not dependent on current political will. The training processes associated with the GRAPH network outputs (data analysis, reporting) and adapted to the needs of local health institutions seek to be extended to academic environments that generate cohorts of young professionals with the skills needed in the public health/epidemiology sector. The network also proposes to assist African Ministers of Health in the implementation of an open source, customizable IT tool for outbreak response (EpiGRAPH hub), that allows interoperability ensuring the enhancement of surveillance systems already in place. Our model promotes autonomy and ownership for end-users/countries in further developing and adapting the tool to their needs which should motivate wider adoption and build capacity beyond this tool.

The GRAPH network is not the unique initiative proposing IT solutions for data management for outbreak response. However The EpiGraph Hub finds itself unique among an ecosystem of related initiatives, mainly due to its bottom-up “collaboration” approach to tool development, heavy focus on capacity building, advanced data infrastructure allowing for integration of heterogeneous data types for state-of-the-art epidemiological analyses, and the maturity of our relationships with the health ministries in a large number of African countries. DHIS2 and SORMAS, both focus on digital health data flows. DHIS2 [28] tools are widely used across LMICs including Africa for data collection, but our in-country members attest that the analyses offered have not been particularly adapted to their epidemic response needs. Furthermore, our system does not require data to be transferred to an external server – it can actually stay in-country at all times. SORMAS [29] appears to have a similar data management and analysis platform in mind, but it is not clear whether population-level analysis rather than digital health data collection is their current focus, nor that they have the same bottom-up approach as we do. So far, we have entered positive partnering discussions with complimentary initiatives such as The R Epidemics Consortium (RECON), Global.health, and openEIMIS.

## Conclusion

The lessons from the initial GRAPH network project, and the positive experiences of researchers during its implementation evidence that it is likely that this global collaborative approach will remain to be a vital part of data management solutions for global health emergencies and outbreaks in the near future. This international collaboration for outbreak response in sub-Saharan Africa also revealed the urgent need to develop projects that focus on local human capacity training and research capacity development. The documentation of the experience gained by implementing the GRAPH network, has also formed a basis for its future development and expansion, experiences that can be implemented in other countries. The GRAPH network is held to be a resource that can be used by all countries implementing similar programs.

In terms of long term sustainability, the GRAPH network aims to contribute to the reinforcement of national surveillance and public health emergency response systems in partner countries in Africa and ultimately the Global South. We aim to develop this project into an operational epidemic response mechanism in which local members increasingly take on leadership roles, ensuring long-term sustainability.

## Supplementary Information

**Additional file 1.** COVID-19 Epidemiological Report. Mauritius. Example In-depth epidemiological report. One out of 45 developed by the GRAPH network.

**Additional file 2.** Web survey. Survey answered by postgraduate students, in-country experts, and other global health professionals.

**Additional file 3.** EpiGRAPH Hub Concept sheet.

## Data Availability

Data sharing is not applicable to this article as no datasets were generated or analysed during the current study.

## Abbreviations

COVID-19: Corona Virus Disease 2019
WHO AFRO: World Health Organization African Region
IHR 2005: International Health Regulations 2005
COVIDSET: Conference of Vice Chancellors and Deans of Science, Education, Engineering and Technology
HIM-WHO AFRO: Health Emergency Information and Risk Assessment - World Health Organization Africa
UNIGE: University of Geneva
ASP: Association Actions en Santé Publique
GRAPH Network: The Global Research and Analyses for Public Health Network
ECDC: European Centre for Disease Prevention and Control
MOH: Minister of Health
DSV: Data Summarization and Visualization tool
LMICs: Low and Middle Income Countries

## References

[1] Impouma B, Roelens M, Williams GS, Flahault A, Codeço CT, Moussana F (2020). Measuring timeliness of outbreak response in the World Health Organization African region, 2017–2019. Emerg Infect Dis.

[2] WHO Africa (2016). Mapping the risk and distribution of epidemics in the WHO African region. A technical report.

[3] Heymann DL, Chen L, Takemi K, Fidler DP, Tappero JW, Thomas MJ (2015). Global health security: the wider lessons from the west African Ebola virus disease epidemic. Lancet.

[4] WHO Africa (2016). Provisional agenda item 8 REGIONAL STRATEGY FOR HEALTH SECURITY AND EMERGENCIES 2016–2020 report of the secretariat.

[5] Emergency response framework (ERF), 2nd edition [Internet]. [cited 2021 May 12]. Available from: https://www.who.int/publications/i/item/emergency-response-framework-(-erf)-2nd-ed

[6] Impouma B, Mlanda T, Bukhari A, Williams GS, Farham B, Wolfe C, et al. Information management practices in the WHO African region to support response to the COVID-19 pandemic. Epidemiol Infect. 2021:1–17 [cited 2021 Jun 11]. Available from: https://www.cambridge.org/core/product/identifier/S0950268821001242/type/journal_article. 10.1017/S0950268821001242 PMC8712935 34036928

[7] Impouma B, Wolfe CM, Mboussou F, Farham B, Saturday T, Pervilhac C, et al. Monitoring and evaluation of COVID-19 response in the WHO African region: challenges and lessons learned. Epidemiol Infect. 2021; [cited 2021 Jun 11]; Available from: https://pubmed.ncbi.nlm.nih.gov/33849676/. 10.1017/S0950268821000807 PMC8723986 33849676

[8] Health workforce [Internet]. [cited 2021 Apr 12]. Available from: https://www.who.int/health-topics/health-workforce#tab=tab_1

[9] WHO Media Center. Global health workforce shortage to reach 12.9 million in coming decades [Internet]. [cited 2021 Apr 12]; Available from: https://apps.who.int/mediacentre/news/releases/2013/health-workforce-shortage/en/index.html

[10] Global Health Workforce statistics database [Internet]. [cited 2021 Apr 12]. Available from: https://www.who.int/data/gho/data/themes/topics/health-workforce

[11] Ngongalah L, Wepngong Emerson coreafricaorg, Niba Rawlings N, Muleme Musisi J. Research challenges in Africa – an exploratory study on the experiences and opinions of African researchers. [cited 2021 Mar 13]; Available from: 10.1101/446328

[12] How much does your country invest in R&D? [Internet]. [cited 2021 Apr 12]. Available from: http://uis.unesco.org/apps/visualisations/research-and-development-spending/

[13] The GRAPH Network [Internet]. [cited 2021 Apr 12]. Available from: https://www.thegraphnetwork.org/

[14] Renku [Internet]. [cited 2021 Apr 12]. Available from: https://renkulab.io/

[15] Iterate faster, innovate together | GitLab [Internet]. [cited 2021 Apr 12]. Available from: https://about.gitlab.com/

[16] GADM [Internet]. [cited 2021 Apr 12]. Available from: https://gadm.org/

[17] Gridded Population of the World (GPW), v4 | SEDAC [Internet]. [cited 2021 Apr 12]. Available from: https://sedac.ciesin.columbia.edu/data/collection/gpw-v4

[18] Git Large File Storage (LFS) | GitLab [Internet]. [cited 2021 Apr 12]. Available from: https://docs.gitlab.com/ee/topics/git/lfs/

[19] GRAPH Network training portal – Training the next generation of public health data analysts [Internet]. [cited 2021 Jun 11]. Available from: https://thegraphnetwork.training/

[20] Ahmed K, Bukhari MA, Mlanda T, Kimenyi JP, Wallace P, Lukoya CO, et al. Novel approach to support rapid data collection, management, and visualization during the COVID-19 outbreak response in the world health organization African region: development of a data summarization and visualization tool. JMIR Public Health Surveill. 2020;6(4) [cited 2021 Apr 3]. Available from: /pmc/articles/PMC7593858/. 10.2196/20355 PMC7593858 32997641

[21] Goldacre B, Harrison S, Mahtani KR, Heneghan C. WHO consultation on data and results sharing during public health emergencies. Background briefing. Sep 2015 Centre for Evidence-Based Medicine Background Briefing: WHO consultation on data and results sharing during public health emergencies background briefing for WHO consultation on data and results sharing during public health emergencies. 2015.

[22] Umviligihozo G, Mupfumi L, Sonela N, Naicker D, Obuku EA, Koofhethile C (2020). Sub-Saharan Africa preparedness and response to the COVID-19 pandemic: a perspective of early career African scientists. Wellcome Open Res.

[23] Confraria H, Blanckenberg J, Swart C (2020). Which factors influence international research collaboration in Africa?.

[24] Keusch GT, McAdam K, Cuff PA, Mancher M, Busta ER. Integrating clinical research into epidemic response: the Ebola experience: Washington, DC: The National Academies Press; 2017. p. 1–316. Available From: https://www.ncbi.nlm.nih.gov/books/NBK441679/28696651

[25] Bryant JH, Harrison PF, Health I of M (US) B on I (1996). Health Research: essential link to equity in development.

[26] Heymann DL, Liu J, Lillywhite L (2016). Partnerships, not parachutists, for Zika research. N Engl J Med.

[27] Only a bottom-up approach will deliver global health development targets [Internet]. [cited 2021 Apr 3]. Available from: https://theconversation.com/only-a-bottom-up-approach-will-deliver-global-health-development-targets-66079

[28] Dhistance [Internet]. [cited 2021 Apr 12]. Available from: https://dhistance.com/?gclid=CjwKCAjwpKCDBhBPEiwAFgBzj8xRTimfTzGQ3Hn1slxx9BJTE-C8nhfjQkaN9UxeeAKux_aK6kFbkxoCO20QAvD_BwE

[29] About – SORMAS [Internet]. [cited 2021 Apr 12]. Available from: https://sormas.org/about

