# Supplementary material for: Leveraging human resources for outbreak analysis: lessons from an international collaboration to support the sub-Saharan African COVID-19 response
Source: BMC Public Health. 2022 May 31;22:1073. doi: 10.1186/s12889-022-13327-1 (PMC9152815; doi:10.1186/s12889-022-13327-1)
Supplement: Supplementary file 1 — Additional file 1. COVID-19 Epidemiological Report. Mauritius. Example In-depth epidemiological report. One out of 45 developed by the GRAPH network. [file 12889_2022_13327_MOESM1_ESM.pdf]

COVID-19 EPIDEMIOLOGICAL REPORT

Mauritius

October, 2020

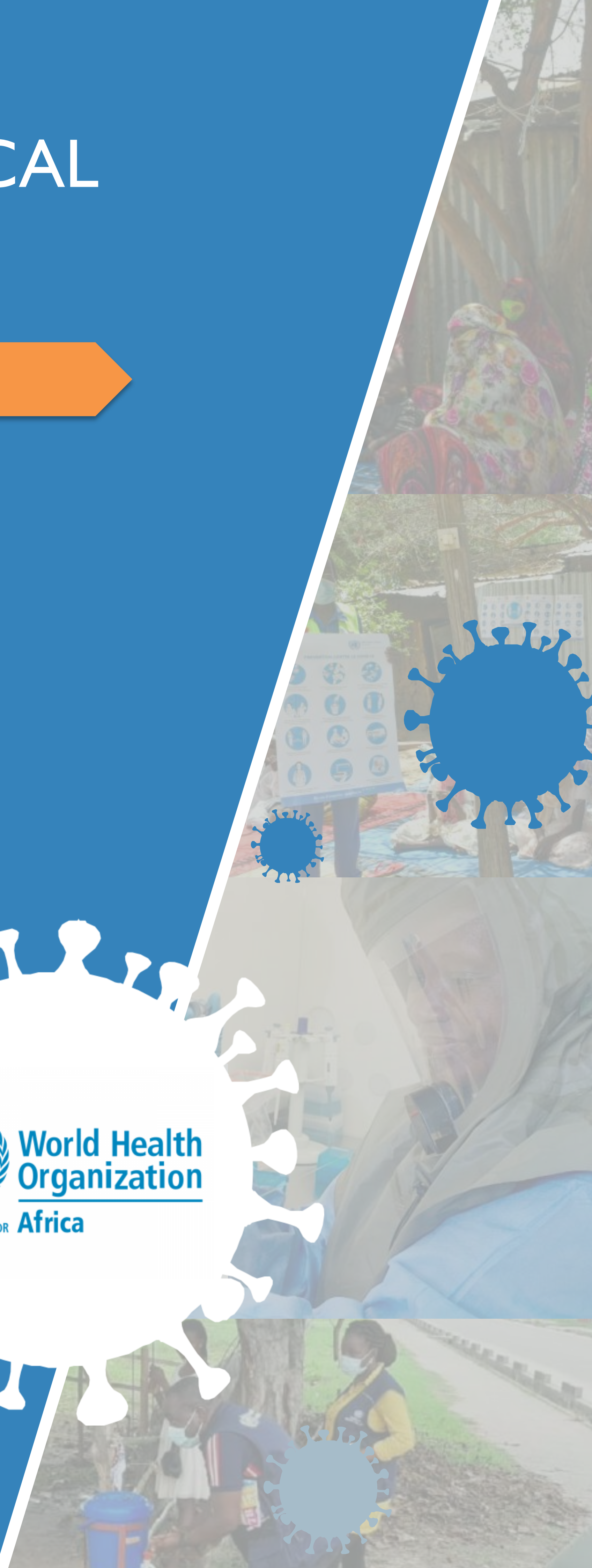

World Health Organization
REGIONAL OFFICE FOR Africa

1. Situation Overview 3
1.1 Timeline 3
1.1.1 Deaths from COVID-19 3
1.2 Response 4
1.3 Situation Update 7
1.3.1 Cumulative Cases and Deaths Per Million 7
2. Detail 10
2.1 Regional analysis 10
2.1.1 Deaths Overview 10
2.1.2 Cases And Deaths Per Capita 12
2.2 Transmission Risk Index 14
2.3 Mortality Risk Index 14
2.4 Age-sex & occupational distribution 15
2.4.1 Age Distribution of Cases Over Time 15
2.4.2 Sex Distribution of Cases Over Time 17
2.4.3 Healthcare Workers 18
3. PHSMs 20
3.1 Further public health and social measures 20
3.2 Adherence to measures 21
4. Issues Affecting the Response 23
4.1 Socio-political And Economic challenges 23
4.1.1 Economic challenges 23
4.1.2 Socio-political challenges 23
4.1.3 Food Security 23
4.1.4 Gender-based Violence 23
5. Interpretation & Recommendations 25
5.1 Situation Summary 25
5.2 Short-Term Action 25
5.3 Long-Term Action 26
6. Appendix 28
7. Acknowledgements 34

1. Situation Overview

(Data updated 4 October 2020)

1. Situation Overview

1.1 Timeline

As of 4 October 2020, Mauritius reported a cumulative total of 395 COVID-19 cases and 10 deaths. This includes 52 new cases confirmed by the authorities since the last epidemiological report on data ending 16 July 2020, Figure 1. Most of the new cases have been reported since the beginning of September, Figure 1.

On 20 March 2020, the Prime Minister of Mauritius confirmed the country's first 3 COVID-19 cases[1]. All three cases were citizens who had recently returned to the country[2] and were immediately placed in isolation at the New Souillac Hospital which was initially identified as the reference treatment center for COVID-19[1]. In response, Mauritius closed its international borders and all educational facilities the following day[1].

Figure 1: Daily COVID-19 incidence in Mauritius including the 7-day rolling average, as of 04 October 2020.

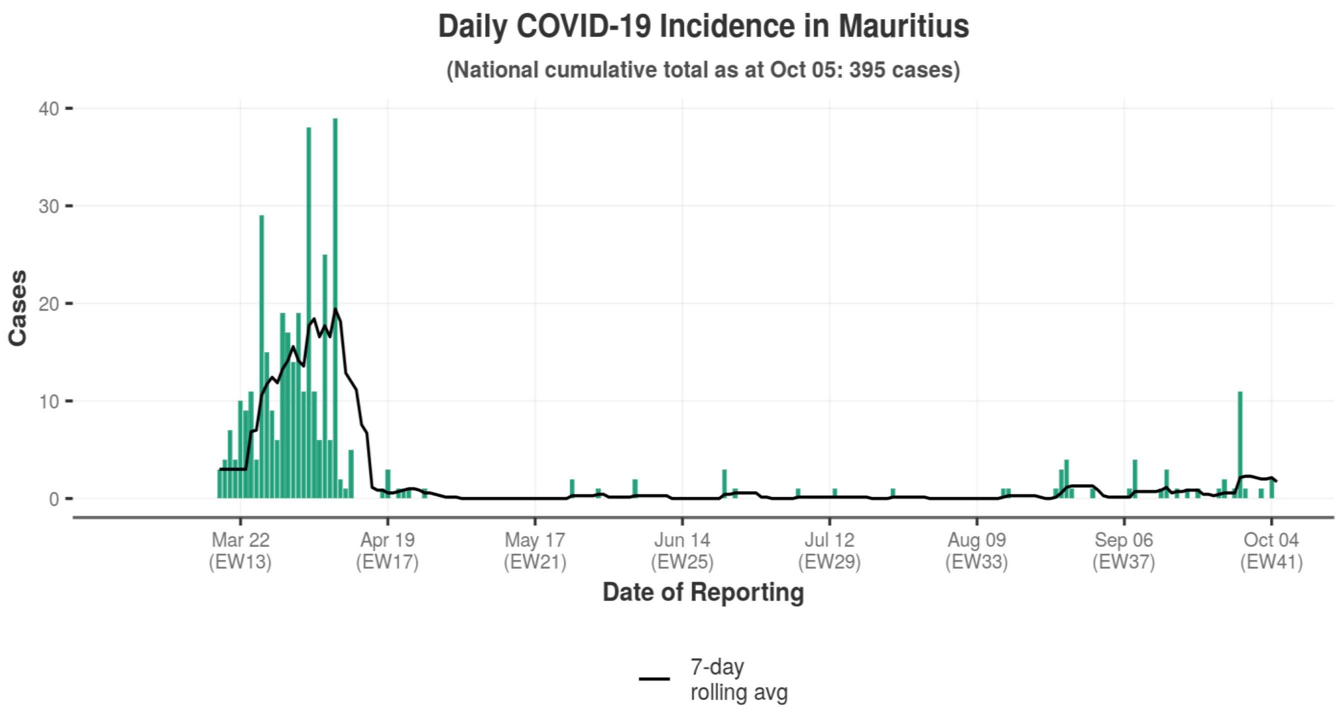

1.1.1 Deaths from COVID-19

Mauritius reported the first death from COVID-19 on 18 March 2020[1]. The cumulative number of deaths as of 04 October 2020 was 10. Both the case count and death rate remained low, Figure 2. The number of deaths remained unchanged since the last epidemiological report on data ending 16 July 2020, Figure 2.

Figure 2: Daily COVID-19 deaths in Mauritius including the 7-day rolling average, as of 04 of October 2020.

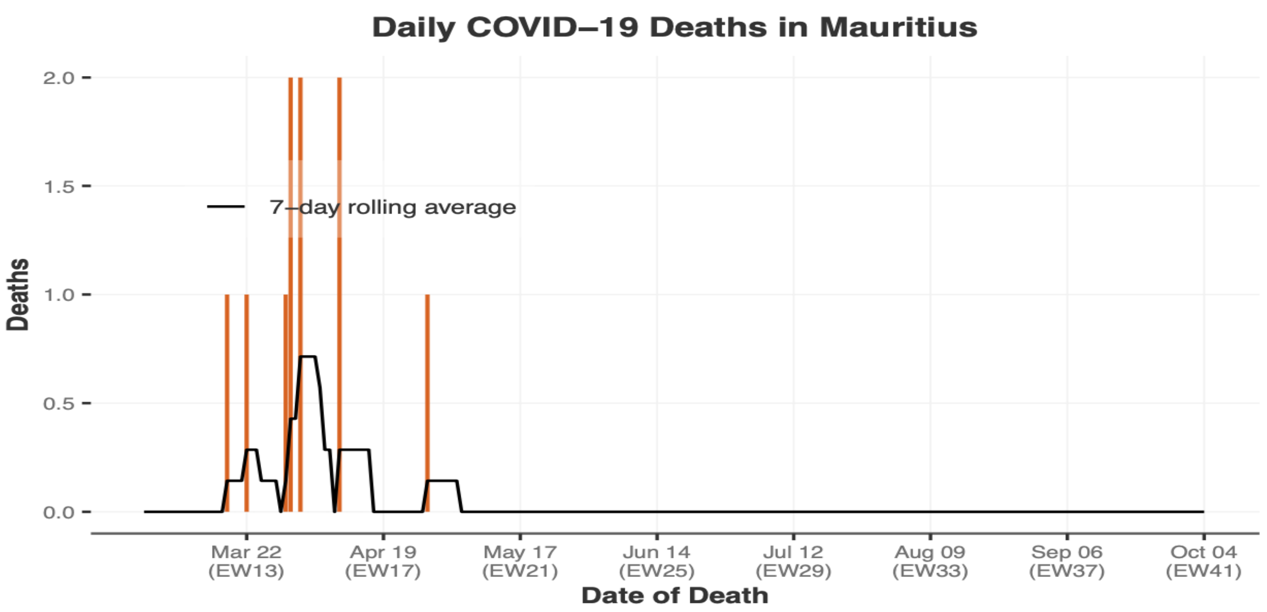

1.2 Response

The country implemented preparedness and prevention measures as early as 20th January 2020 while the outbreak was declared late March 2020. These measures included a.) screening all passengers in all the ports and harbours of the country with thermal scanners and b.) monitoring of passengers by health professionals for a period of 14 days starting from the day of their arrival[1]. From the 31 January, all direct flights from China were suspended and as the virus spread to additional countries, the regulation was extended to include South Korea, Italy and Iran as well. Simultaneously, the health authorities expanded the available quarantine facilities[1] as 11 recreational centres and several hotels were converted in order to provide a total of 35 quarantine facilities. In addition, on the 22 March, 3 hospitals were converted to accommodate COVID-19 cases and one facility dedicated for more serious cases. Also, 14-seater vehicles were converted to ambulances for COVID-19 response, in conjunction with the ambulances belonging to private medical facilities[2,5].

The country closed its international borders and all educational institutions on 19 March. The 20th of March 2020 was the first day of national lockdown, with a curfew implemented on 23 March until 31 May 2020, however, essential services and food markets continued their operation. People were allowed to shop twice per week based on an alphabetical schedulewhere customers were allotted 30-minutes purchasing period inside the store. The use of a mask and social distancing were obligatory. There was also an online service where people could order essential goods, operating until the end of the lockdown[1].

1. Goal, Mauritius terminates football season, 2020. Retrieved from https://www.goal.com/en/news/coronavirus-mauritius-terminates-football-season/1wz0obtlbt7js1uwi2y7xjd43x
2. Republic of Mauritius, COVID-19 Communiqués 2020. Retrieved from http://www.govmu.org/English/Pages/ViewAllCommuniquecovid19.aspx
3. UNDP (2020), Support to the National Response to Contain the Impact of COVID-19. Retrieved by https://www.undp.org/content/dam/rba/docs/COVID-19-CO-Response/Government_COVID_Responses_Mauritius_Seychelles_29March2020RB-2.pdf
4. Government Information Service, Prime Minister's Office (September, 2020) COVID-19: Mauritius to reopen borders in three phases Retrieved from http://www.govmu.org/English/News/Pages/COVID-19-Mauritius-to-reopen-borders-in-three-phases-.aspx
5. Republic of Mauritius, News, 22 May 2020. Retrieved from: http://www.govmu.org/English/News/Pages/default.aspx?NewsD=Health

The government implemented a series of surveillance measures aimed to manage the outbreak. They isolated all confirmed cases in dedicated medical facilities. In addition to isolation, the healthcare personnel traced all contacts of the positive cases[1]. In this regard a COVID-19 testing center was set up in each of the five public regional hospitals of the country[15]. The construction of the testing centers was funded by the Ministry of Health and Wellness, Private sector, UNDP and WHO. Mass screenings were performed since 27 April, especially targeting frontline staff including healthcare personnel, members of the police force, the special mobile force, the National Guard force, the prison staff, and supermarket and shop employees. By the 22 May, around 8% of the population had been screened[5].

As the measures managed to contain the transmissions, the government decided to resume some of the financial activities from 15 May 2020. To this end, the authorities provided proper authorisation to all workers who needed to return to duties such as caretakers, babysitters, fishermen, and planters. The educational facilities restarted in August 2020. In addition, the plan included provisions for the elderly as they were the most vulnerable groups, namely at home medical visits, distribution of pension at homes and special desks in the banks[6].

On 29 May 2020, the Prime Minister announced that most of the restrictions were lifted as of 30 May 2020, and several economical activities would resume normally. There are still some measures and restrictions in place that all citizens need to follow, to avoid a future surge of cases. From 31 May 2020, citizens would be allowed to go out, no work permit authorisation would be needed, nurseries and daycare centers would restart operating, as well as super markets and stores like bars, shopping malls, restaurants. All people were obliged to use masks and maintain social distancing in public places. In addition, people would be allowed to visit places of worship and resume individual sporting activities. Only nightclubs, beaches and cinemas would remain close[7].

At the apex of the response mechanism, a high level committee on COVID-19 was instituted and chaired by the Prime Minister. The committee also comprised of several ministers (Health and Wellness, Foreign Affairs, Finance, Local Government, Tourism), WHO Representative and Commissioner of Police. A whole-of-government approach enabled timely strategic informed decision making for a coordinated and scaled up national response. A National Communication Committee headed by the Prime Minister was also set up and various stakeholders within the committee made up the communication strategy, The designated spoke person of the committee ensured daily communication with the public on the national TV and radio[1]. A YouTube channel (https://www.youtube.com/playlist?list=PLbzHFuDxBTGSpOyFenxXRAGEcCh9nt2I3), a Facebook page (https://www.facebook.com/coronavirusmoris/) and an official website (https://covid19.mu/) were set up, aiming to inform and sensitize the population[8]. There was also a hotline available to the public, operating 24 hours every day[10]. Furthermore, in collaboration with Mauritius Telecom, the government launched the app called

6. Government Information Service, Prime Minister's Office (May, 2020) Covid-19 Bill will provide necessary legal impetus to enforce post-lockdown measures Retrieved from http://www.govmu.org/English/News/Pages/Covid-19-Bill-will-provide-necessary-legal-impetus-to-enforce-post-lockdown-measures.aspx
7. Government Information Service, Prime Minister's Office (May, 2020) Covid-19 Mauritius to lift curfew on 30 May 2020 Retrieved from http://www.govmu.org/English/News/Pages/Covid-19-Mauritius-to-lift-curfew-on-30-May-2020,-at-midnight,-says-PM.aspx
8. Republic of Mauritius Portal, Ministry of Health and Wellness, 2020. Retrieved from: http://health.govmu.org/English/Pages/covid%20-19.aspx
9. UNDP, Mapping of Fiscal and Social Protection Policy Responses in Mauritius and Seychelles, 2020. Retrieved by https://www.undp.org/content/dam/rba/docs/COVID-19-CO-Response/Government_COVID_Responses_Mauritius_Seychelles_29March2020RB-2.pdf
10. Ministry of Health and Quality of Life, Government of the Republic of Mauritius, Health 2015 – Seamless Continuity of Care, 2015. Retrieved from https://www.who.int/goe/policies/mauritius_health2015.pdf?ua=1
11. Government Information Service, Prime Minister's Office (September, 2020) COVID-19: Mauritius to reopen borders in three phases Retrieved from http://www.govmu.org/English/News/Pages/COVID-19-Mauritius-to-reopen-borders-in-three-phases-.aspx
12. Ministry of Health and Wellness (September,2020) Internal Communication Sitrep: COVID-19 Situational Report
13. Ministry of Health and Wellness (June,2020) Internal Communication Sitrep: COVID-19 Situational Report
14. Ministry of Tourism (September, 2020), Opening of borders to all passengers willing to undergo a 14-day quarantine in a state-designated facility Retrieved from https://www.mymauritius.travel/articles/opening-borders-all-passengers-willing-undergo-14-day-quarantine-state-designated-facility
15. Government of mauritius (04 May 2020), Covid-19: Gradual re-opening of businesses after Mauritius marks 14 days without new cases, http://www.govmu.org/English/News/Pages/Covid-19-Gradual-re-opening-of-businessesafter-Mauritius-marks-14-days-without-new-cases.aspx
16. World Health Organisation Country Office Mauritius, Government of Mauritius (October 2020), Best practices and experience of Mauritus prepardness and response to COVID-19 pandemic. Accessed from https://reliefweb.int/sites/reliefweb.int/files/resources/Mauritius%20Inter-Action%20Review%201%20COVID-19%20%20Report.pdf

”beSafeMorris”, providing information about news and communications, health tip videos and health centres, quick access to hotline numbers, notifications and FAQs[10]. Moreover, online consultations were accessible through the "medicine.mu" app to decrease crowding in hospitals and medical facilities[10]. Additionally, the country bought advertisement slots in the media and in the transportation system[9].

Preparation of the Laboratory Services regarding testing practices for COVID-19 has been one of the main areas of focus in the country's response to the outbreak. Since the mid-January, the National Health Laboratory Services started organising the protocols to be followed and purchasing the necessary supplies to conduct COVID-19 tests in the country. PCR testing for COVID-19 on incoming people started in February 2020 and soon after included people identified at high risk from medical professionals. Mauritius adopted its testing strategy with a rate of 180 tests per 1000 population and a total of 89,036 PCR tests by 27 September 2020, by testing people working in the frontline, vulnerable groups and asymptomatic people in high risk. As the need for COVID-19 tests increased rapidly, the National Laboratory partnered with the UNDP Global Center for Technology, Innovation and Sustainable Development to leverage a new digital system which enhanced the efficiency of the whole testing process. In addition, since April 2020 the testing capacity increased by including a private laboratory working in collabarotaion with the National one[16].

Apart from the testing procedures, the Health Authorities ensured good collaboration and coordination between various groups working in the National response plan. More specifically, the personnel working in sample collection, storage and transportation received training from the Central Health Laboratory experts. In addition, there was good communication between the Laboratory and Communicable Diseases Control Unit, so that people with positive tests would be moved to a quarantine center or a hospital in a timely manner. Besides, the up to date information and statistics have been disseminated rapidly among decision makers and public health authorities. It seems that previous experience with disease outbreaks like ebola, zika and MERS-CoV played a central role in the implementation of a comprehensive testing strategy for COVID-19[16].

In the beginning of September, the Prime Minister of Mauritius announced in a televised message, that the reopening of the borders will be carried out in 3 discrete stages. The first phase included the repatriation of stranded Mauritian citizens, which had been ongoing till 30 September 2020. The second phase started from 1 October 2020, during which commercial flights to and from Mauritius were allowed. Priority was given to Mauritian nationals, residents and people with work permit. The third phase which comprises the complete reopening of the borders hasn't been announced yet and will depend on the evolution of the COVID-19 outbreak[4].

The repatriation of stranded citizens from abroad was a priority of the Mauritian Government since the beginning of the outbreak, and has been an ongoing process. The repatriation procedures started even before the lockdown and intensified from June 2020. The National Committee is responsible for all decisions regarding repatriation plans based on the national quarantine and treatment capacities[11]. All Mauritians citizens are allowed to return to the country following a protocol designed by the government which requires a negative PCR test done at most 5 days prior to the flight. All returnees are quarantined in one of the dedicated quarantine facilities upon arrival for 14 days. During this time, the people need to undergo 2 additional PCR tests, one after the first week and an exit screening at the end of the quarantine periods[13]. In addition, the Ministry of Foreign Affairs assembled a repatriation plan for the Mauritians employed by various cruise ships around the globe[11]. As of 1 of October 2020, 8,383 citizens have been repatriated[12].

On the 2 October 2020, the second phase of the border re-opening started, during which people fitting particular criteria would enter the country from specific entry points[3]. This new phase allows Mauritians and non-Mauritians to

visit the country on a 14-day quarantine period in a designated hotel room operating as a quarantine facility, paid by the travellers. After the end of the quarantine, if the tests are all negative, people are allowed to move freely across the country. The Ministry of Tourism has already published a guide which includes detailed information regarding the travellers' journey from the time of the arrival to their departure flight[14].

1.3 Situation Update

Since the beginning of the outbreak in Mauritius, the government implemented strict measures, aiming to contain the spread of the virus. As a result the total number of cases as of 04 October was 395, while the last case of local transmission was reported on the 26th of April 2020. Afterwards, as we can also see on Figure 1, there have only been a few confirmed COVID-19 cases, all of which were imported.

1.3.1 Cumulative Cases and Deaths Per Million (international comparisons)

The cumulative number of COVID-19 cases in Mauritius remained relatively low throughout the outbreak in the country, as shown in Figure 1. Compared to other countries in the region, Mauritius has had a relatively low absolute and per-capita number of cummulative cases (Table 1). Compared to other countries categorized as Small Island Developing States (SIDS) or have a similar development pattern (Maldives and Singapore), Mauritius recorded the lowest per capita number of cases (Figure 3). Furthermore, Mauritius managed to control their epidemic very early on, and has only had sporadic imported cases since April.

Table 1: Case counts, death counts, CFR and per-capita adjusted values in Eastern Africa

Country	Cases	Deaths	Crude CFR	Population	Cases per million	Deaths per million
Kenya	39427	731	1.9	52314849	753.6	14
Ethiopia	78819	1222	1.6	112833693	698.5	10.8
Madagascar	16558	232	1.4	27797000	595.7	8.3
South Sudan	2726	50	1.8	14839454	183.7	3.4
Rwanda	4866	29	0.6	13117385	371	2.2
Uganda	8808	81	0.9	49051105	179.6	1.7
Tanzania	509	21	4.1	64577992	7.882	0.3
Eritrea	398	0	0	5930313	67.11	0
Mauritius	**395**	**10**	**2.6**	**1291483**	**298.1**	**7.7**
Seychelles	142	0	0	98885	1436	0

Figure 3: Per-capita adjusted cumulative COVID-19 cases in Mauritius compared to other countries categorized as Small Island Developing States (SIDS) or have a similar development pattern (Maldives and Singapore), as of 04 of October 2020.

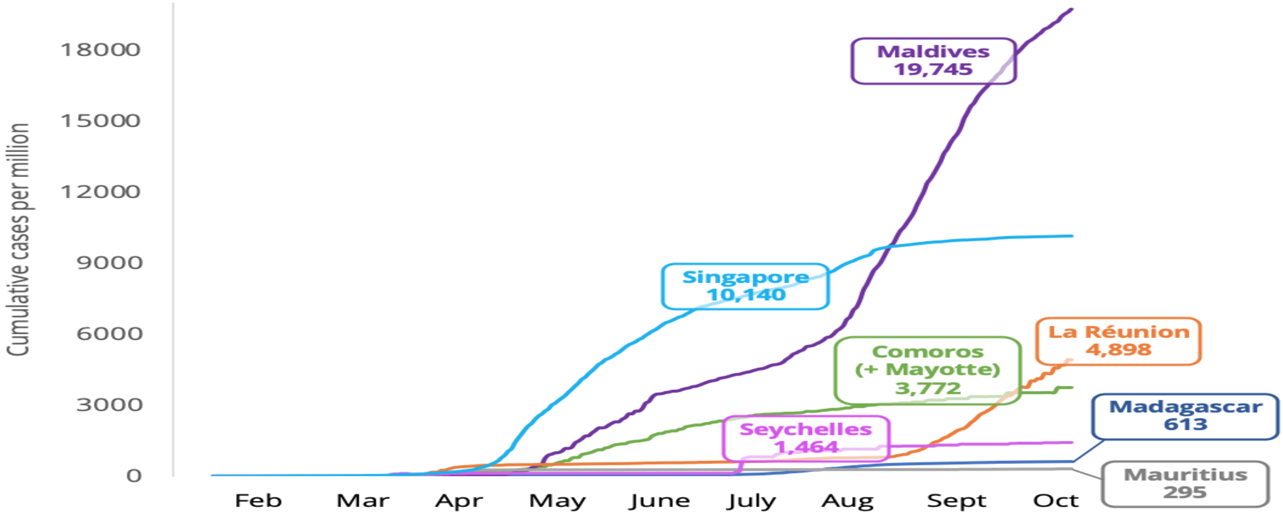

While the total number of deaths (10) was also the lowest for the region (Table 1), both the crude case fatality rate (CFR, 2.6) and per capita number of deaths (7.7) were among the highest in the region. This, despite there being no new deaths since April 2020. However, when compared to the other island nations, it was again among the least affected (Figure 4). Data for Figures 3 and 4 were downloaded from the WHO COVID-19 dashboard at https://covid19.who.int/.

Figure 4: Per-capita adjusted cumulative COVID-19 deaths in Mauritius compared to other countries categorized as Small Island Developing States (SIDS) or have a similar development pattern (Maldives and Singapore), as of 16 of July 2020.

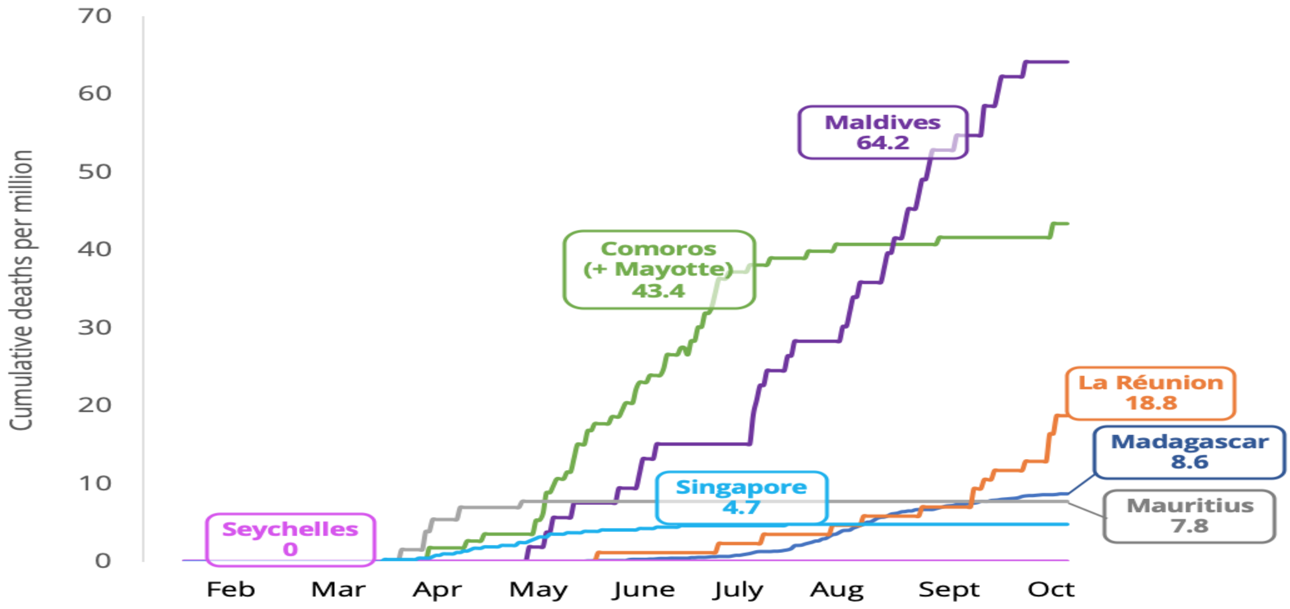

2. Detail

(Data updated 4 October 2020)

2. Detail

While the first section included up-to-date information extracted from MOHW situation reports, this section provides more detailed analyses made possible by patient line-list data provided by the country.

2.1 Regional analysis

The first 3 COVID-19 cases were reported on the 18 March 2020, in the districts of Flacq and Plaine Wilhems. The immediate closure of the international borders prevented additional influx of imported cases. By the 28 March 2020, 9 districts in Mauritius had reported at least one positive COVID-19 case, as shown in Figure 5. Rodrigues island is the only district that has been COVID-free since the beginning of the outbreak.

Figure 5: Daily COVID-19 cases across districts in Mauritius

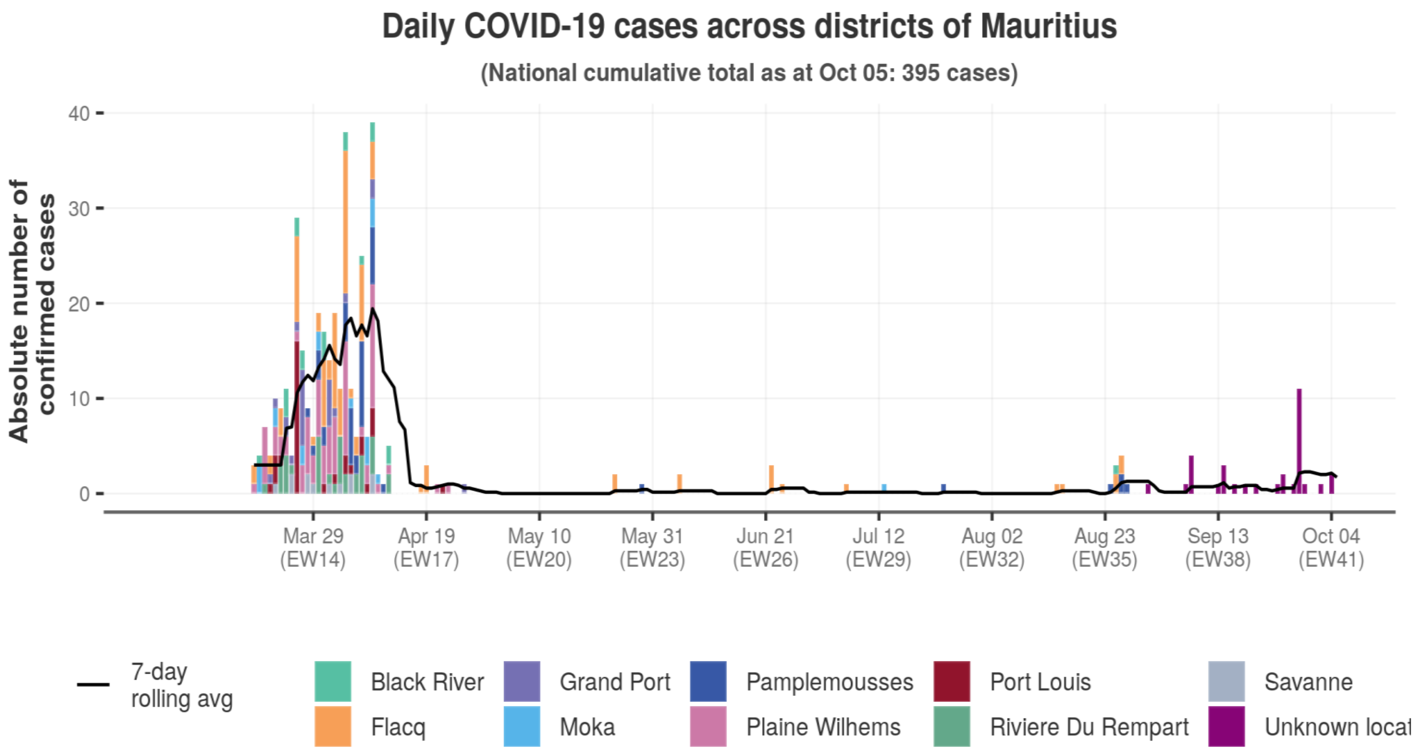

Figure 6: Daily COVID-19 cases across regions in Mauritius in relative numbers

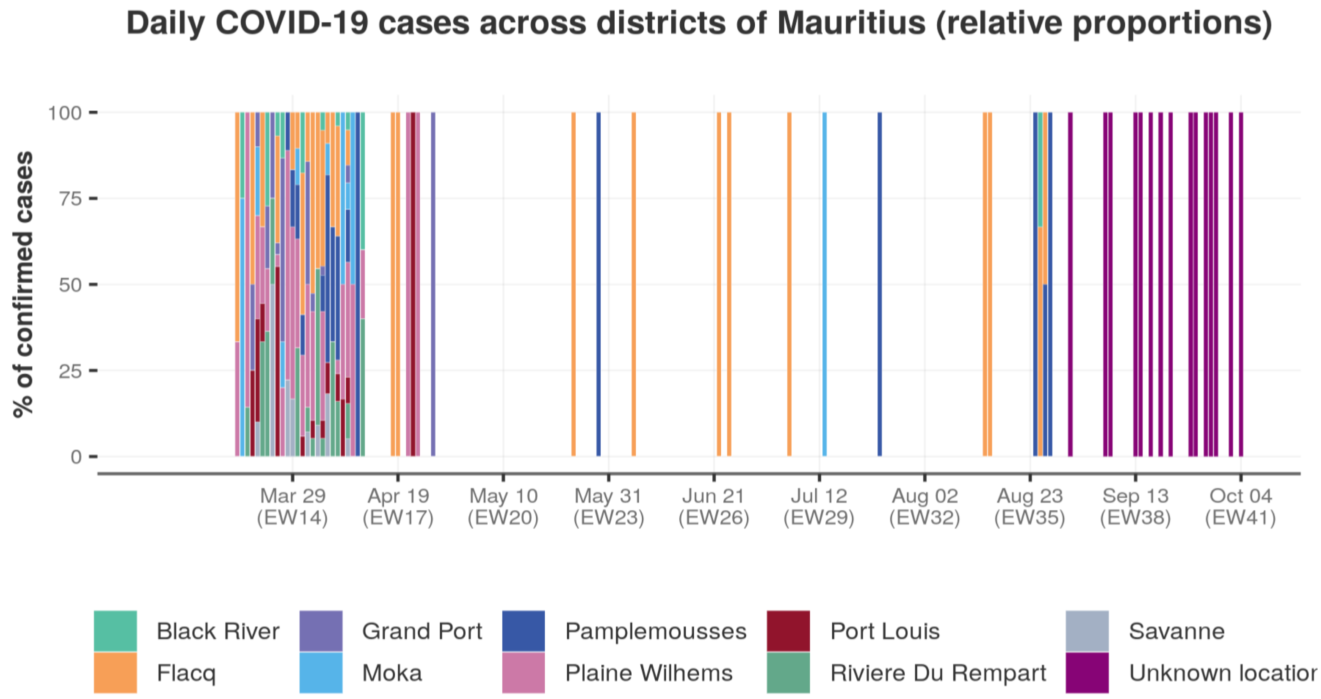

The most affected district in Mauritius appears to be Flacq, with 92 total confirmed cases, which also reported the first confirmed cases in the country. Looking at cases per capita per district, the most affected districts are Flacq and Riviera du Rempart, as shown in Figure 7.

Figure 7: Total confirmed imported and locally acquired cases and cases per million across districts in Mauritius, as of 4 October 2020

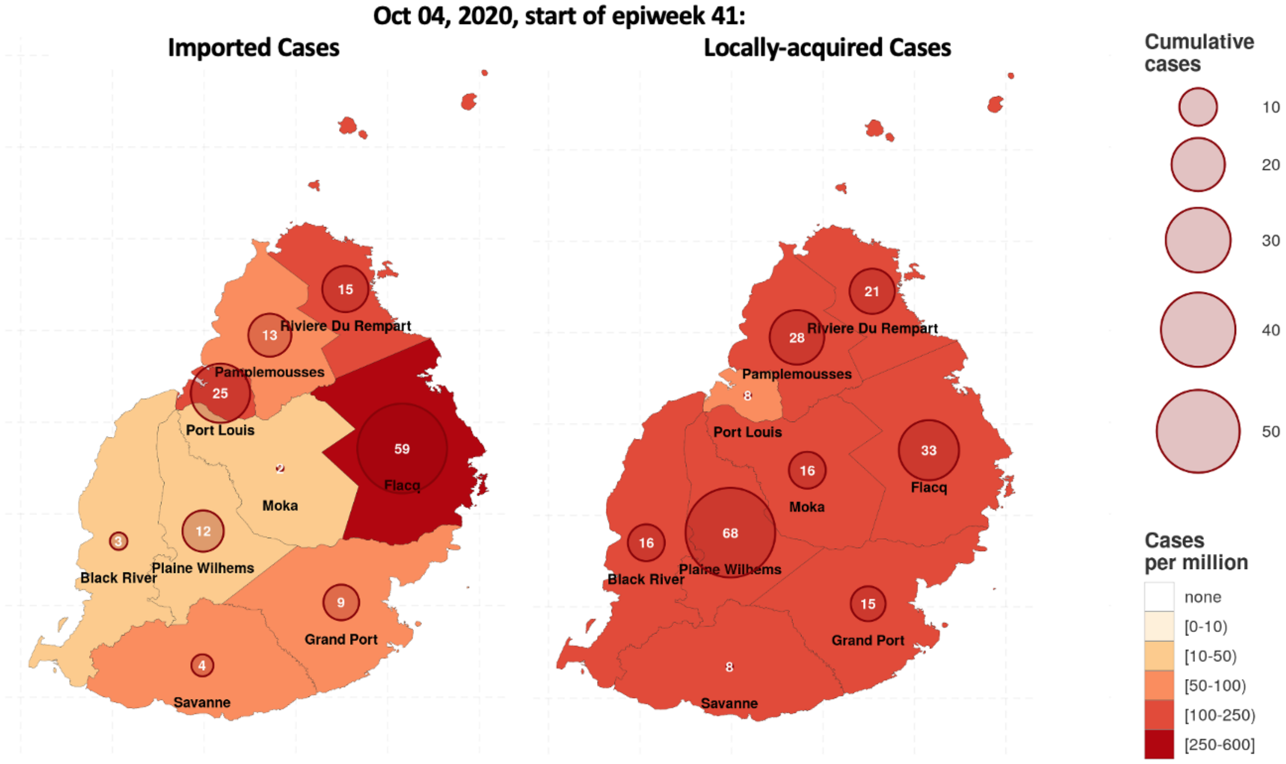

World Health Organization

2.1.1 Deaths Overview

The highest number of deaths was recorded in Plaine Wilhems (4) and the highest CFR was noted in Black River where two people died out of the total confirmed COVID-19 case count of 19 (see Figures 8 and 9). No new deaths have been reported since April 2020.

Figure 8: Daily confirmed COVID-19 deaths across districts in Mauritius

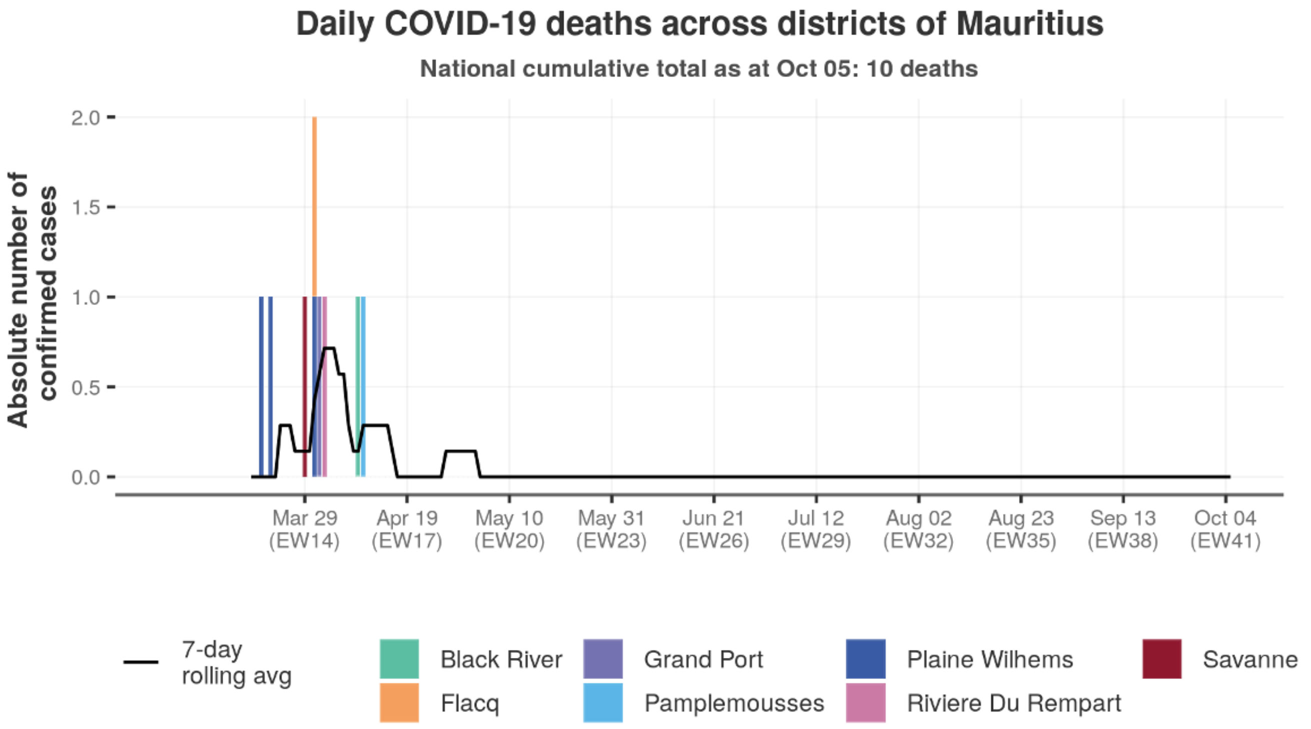

Figure 9: Total cases, deaths and crude CFR across districts in Mauritius

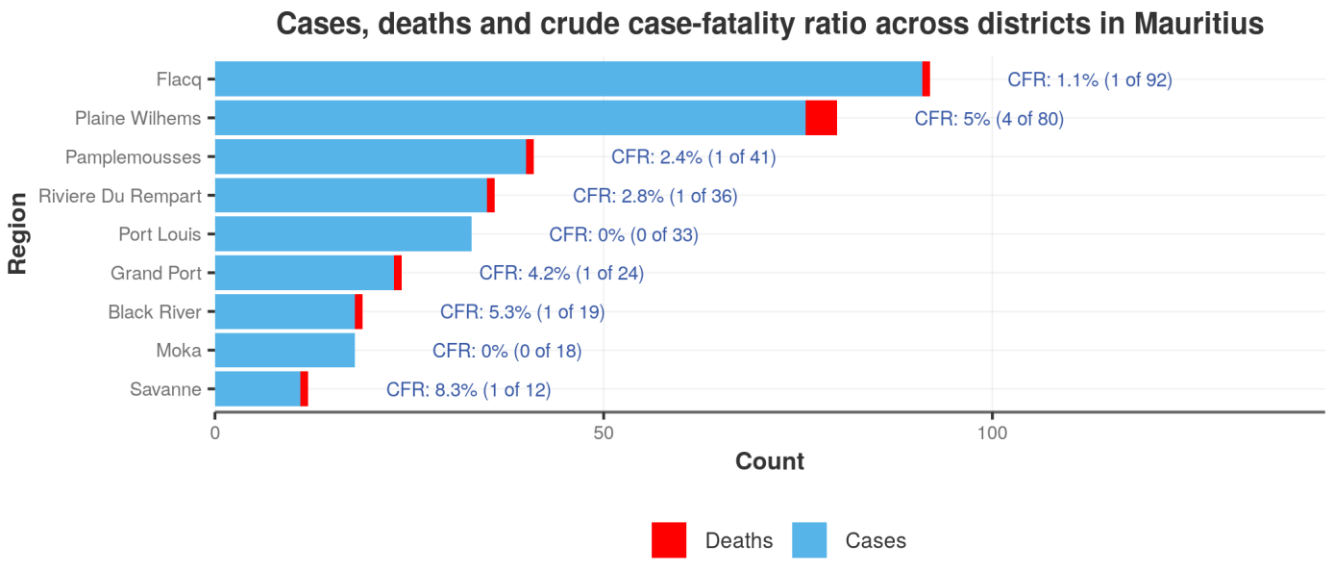

2.1.2 Cases And Deaths Per Capita (Latest Data from 4 Oct 2020)

In absolute numbers, Flacq and Plaine Wilhems are the two districts with the highest number of cases, but when adjusted for population size, Flacq and Riviere Du Rempart were the most affected districts, Figure 10. Regarding deaths per population, Black River and Savanne display higher numbers, Figure 11.

Figure 10: Cumulative cases per million across the top-five regions in Mauritius

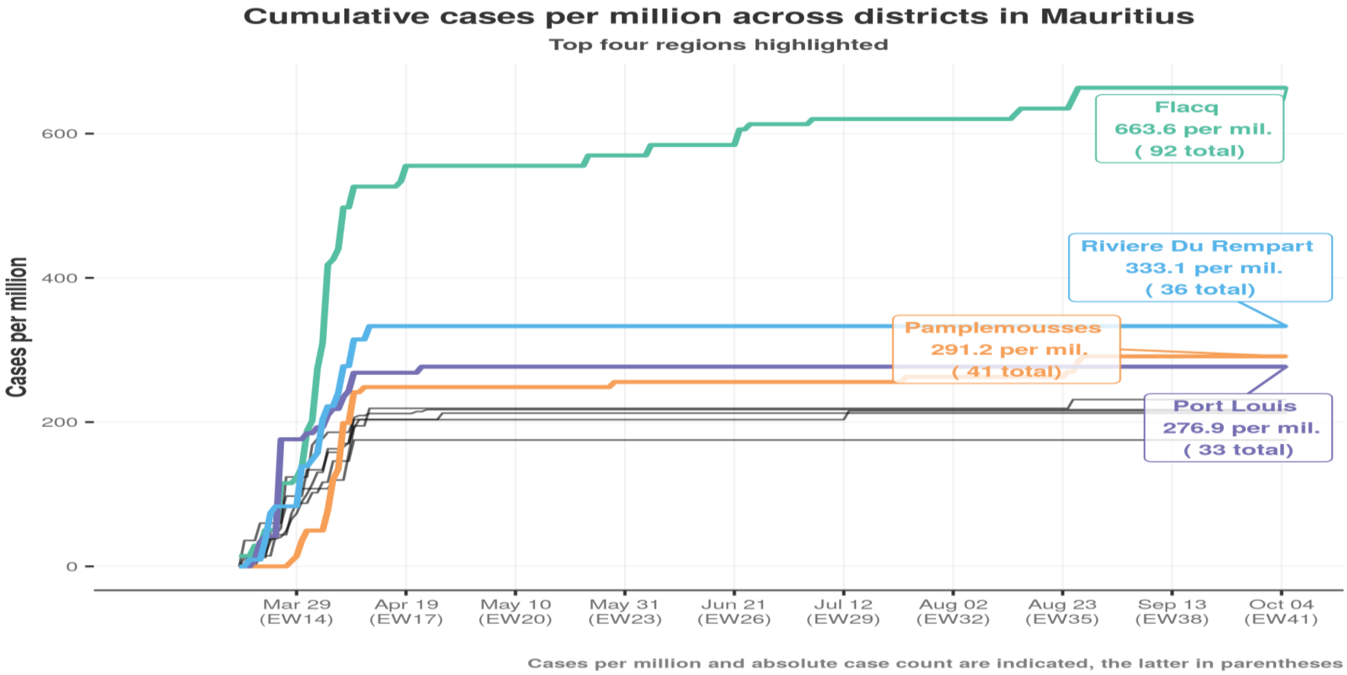

Figure 11: Cumulative deaths per million across the top-five regions in Mauritius

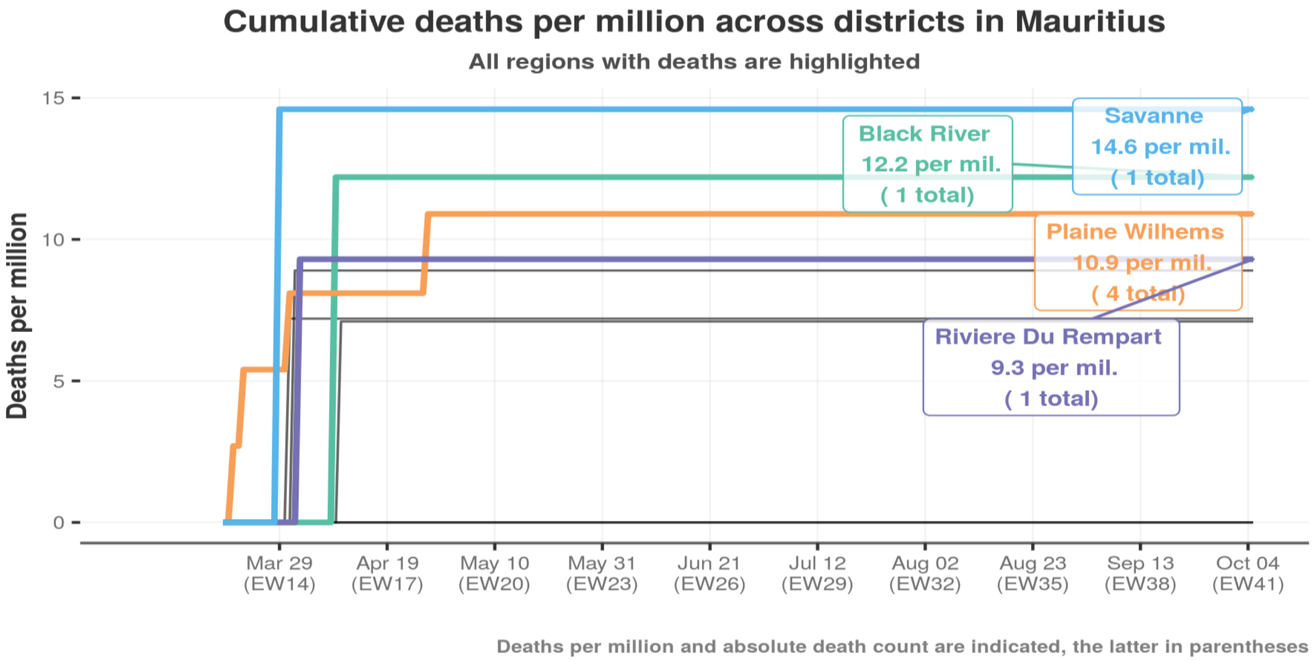

2.2 Transmission Risk Index

Based on the current reported COVID-19 cases and deaths as well as the trend of cases and deaths since the beginning of tracking, Figure 12 below shows the risk of transmission and spread of COVID-19 at the Regional level. The risk rate is relative to other Regions within Mauritius. The filled color tracks the level of risk as defined in the legend. When interpreting the diagram, we need to keep in mind that Mauritius' last local transmission was reported in April.

Figure 12: Mauritius Transmission Risk Index, as of October 4, 2020 – Regional

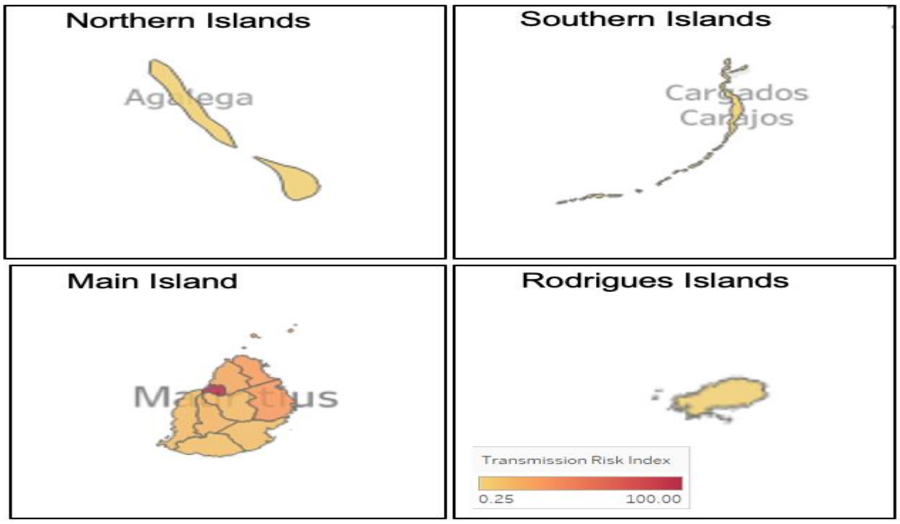

2.3 Mortality Risk Index

Based on the population demographics and underlying health posture of the country, the risk of mortality or of developing a critical case due to COVID-19 is shown on the Figure 13 below at the regional level. The filled color tracks the level of risk as defined in the legend. No new deaths have been reported in Mauritius since the first wave in April 2020.

Figure 13: Mauritius Mortality Risk Index, as of October 4, 2020 – Regional

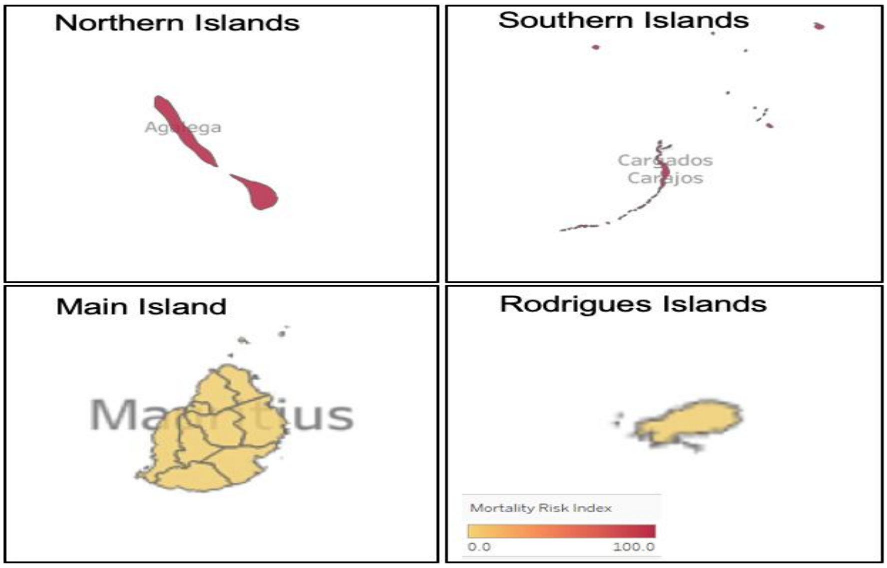

World Health Organization

2.4 Age-sex & occupational distribution

Figure 14 provides an overview of the age – sex distribution of Mauritius' total confirmed COVID-19 cases and deaths, displaying the crude CFR as well, among patients for which data were available. The number of confirmed cases was higher for the age groups of 20-49. This may be explained by the higher exposure of these age groups (working population) and the young age distribution of the Mauritius population, which is consistent the distribution of cases. Additionally, media campaigns focusing on protecting the elderly may have been effective. In terms of sex distribution, the total number of confirmed cases for most age groups was higher for men than for women, with exceptions for kids under 5 years of age, and above 80 years old. There was a higher risk of death for men, as just one of the 10 deaths in the country was a woman (1/171 vs 9/250: $\chi^2 = 3.98$, p = 0.046).

Figure 14: Age sex distribution of the total of confirmed COVID-19 cases and deaths, as of 4 October 2020. The figure is displaying the respective case fatality rates as well.

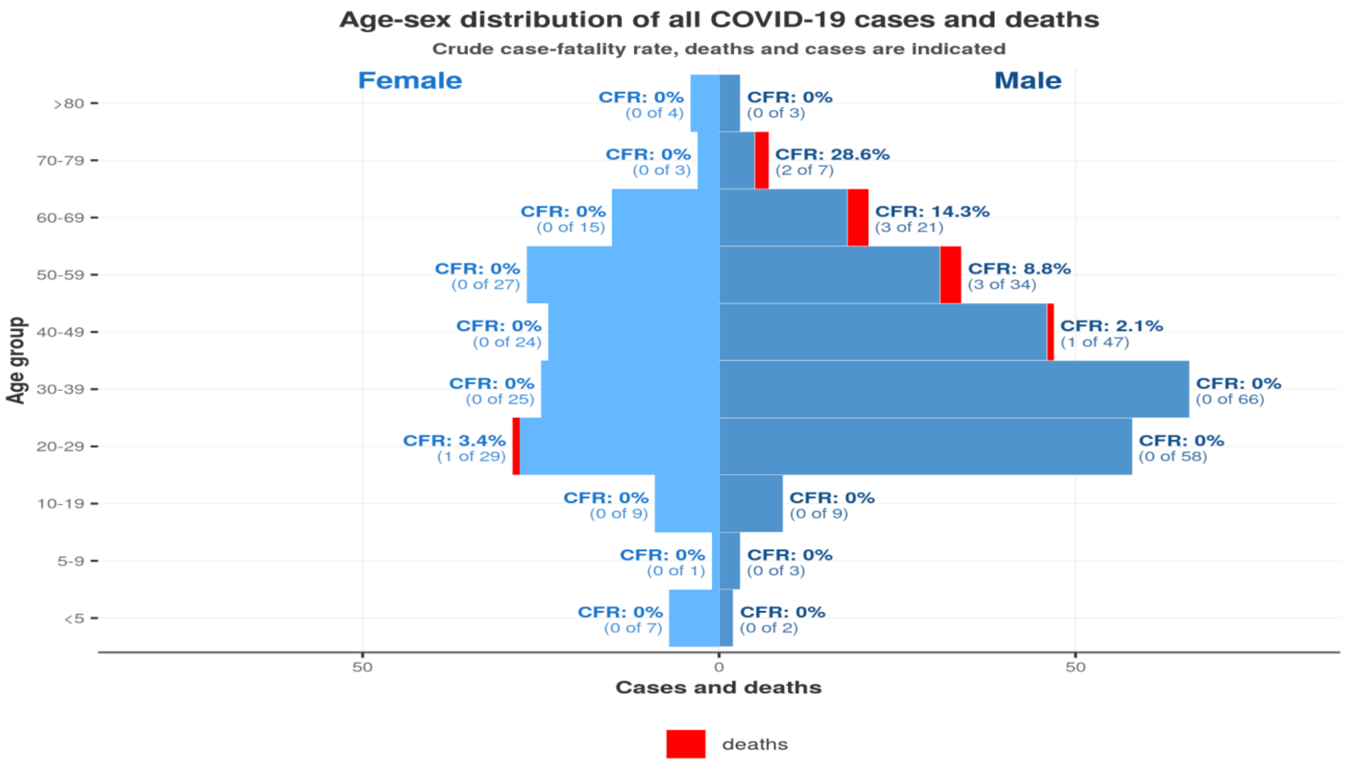

2.4.1 Age Distribution of Cases Over Time

Figures 15 and 16 illustrate the absolute and relative age distribution of cases over time. It is important to note that all cases after April were imported.

Figure 15: Age distribution of new COVID-19 confirmed cases in absolute numbers

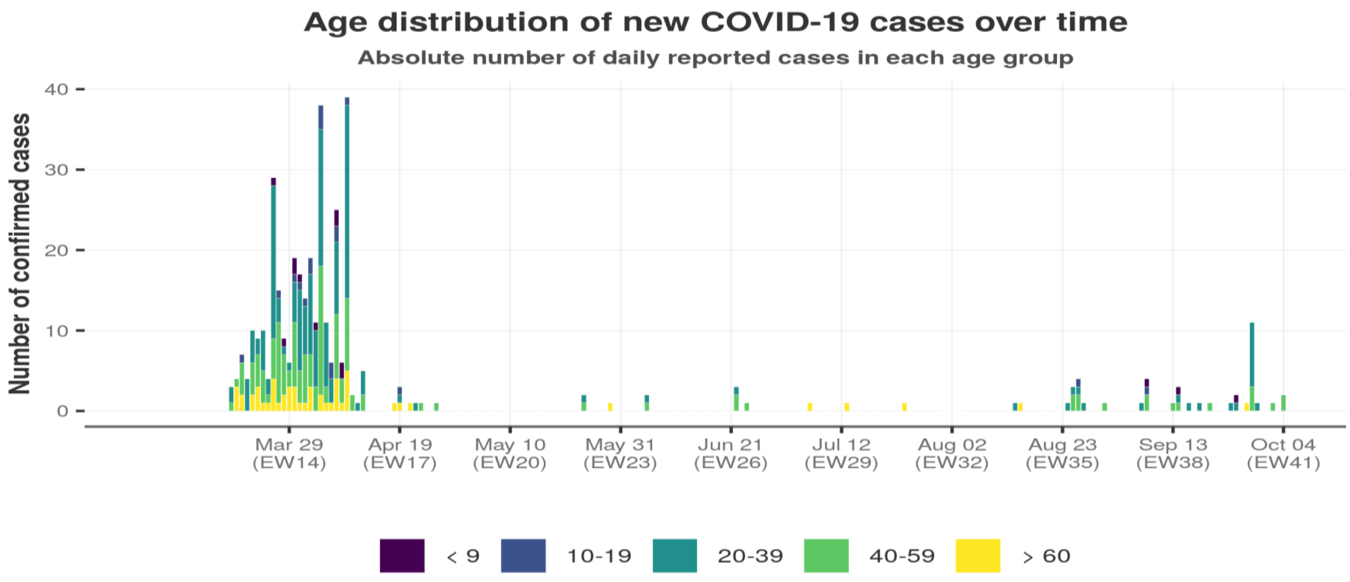

Figure 16: Age distribution of new COVID-19 confirmed cases in relative numbers

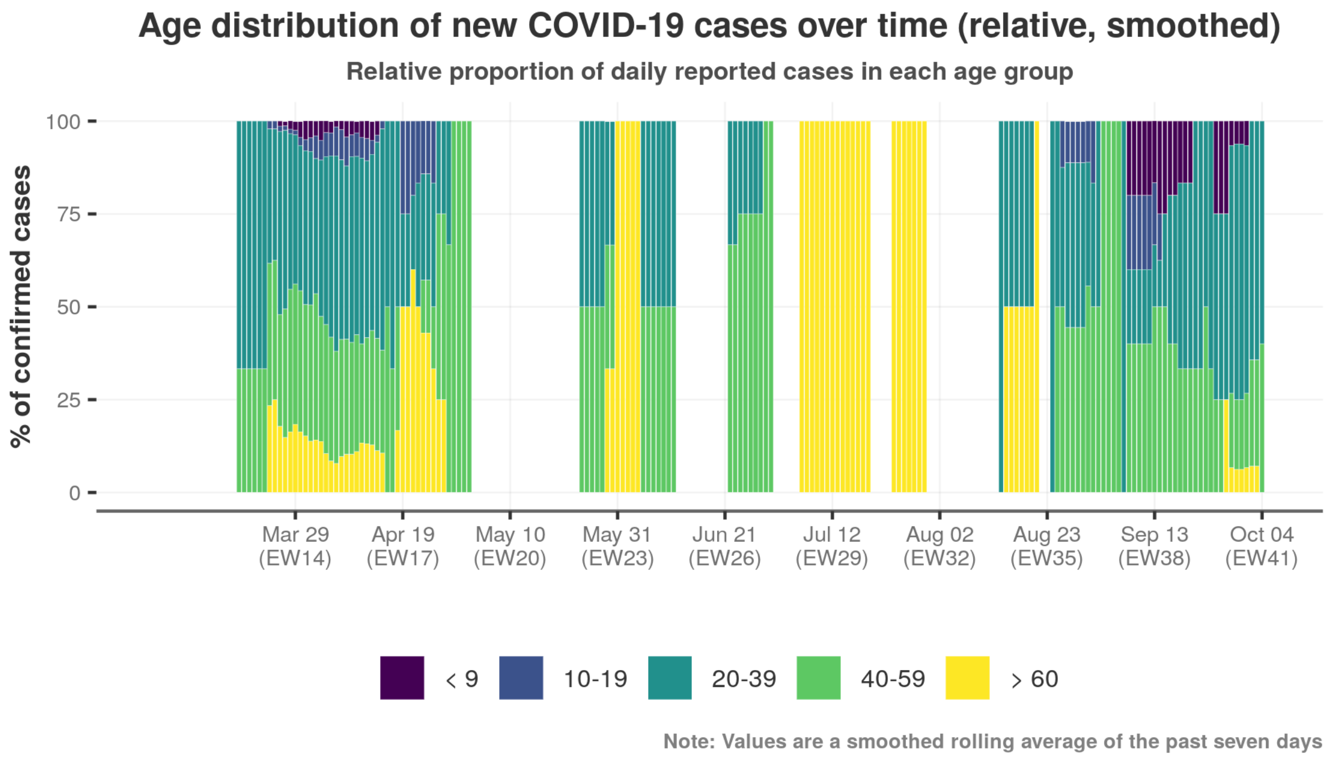

2.4.2 Sex Distribution of Cases Over Time

The absolute and relative sex distribution over time is illustrated in Figures 17 and 18. The majority of cases have been men, perhaps reflecting higher frequency of international travel.

Figure 17: Sex distribution of new COVID-19 confirmed cases in absolute numbers

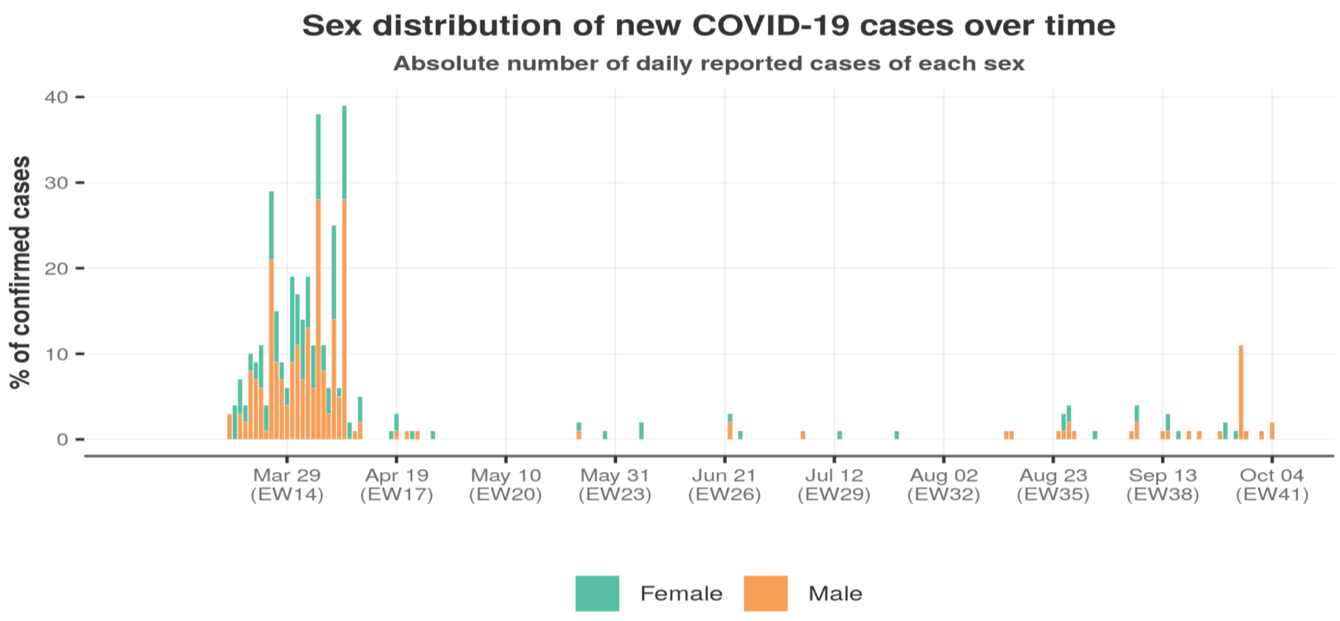

Figure 18: Sex distribution of new COVID-19 confirmed cases in relative numbers

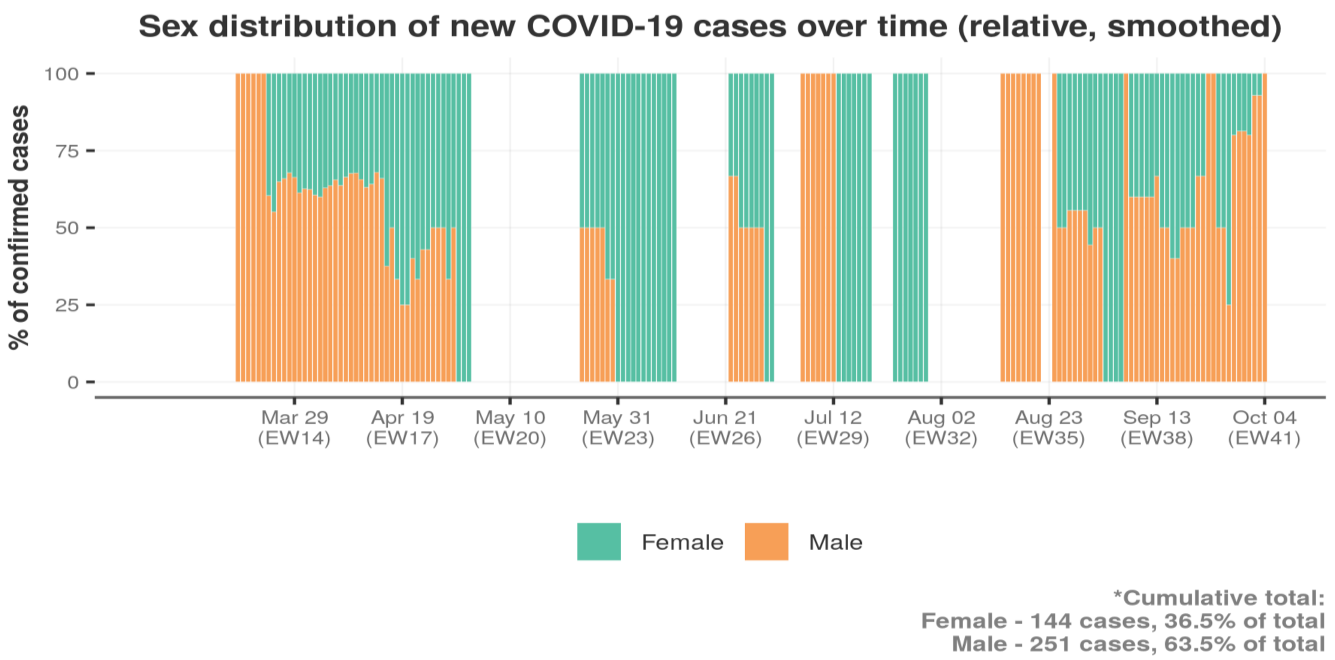

2.4.3 Healthcare Workers

As illustrated in Figure 19, the number of healthcare workers that were infected by SARS-CoV-2 increased significantly two weeks after the initial peak of COVID-19 in the country. Since the previous epidemiological report, no new healthcare workers have been infected.

Figure 19: Daily COVID-19 cases in healthcare vs non-healthcare workers

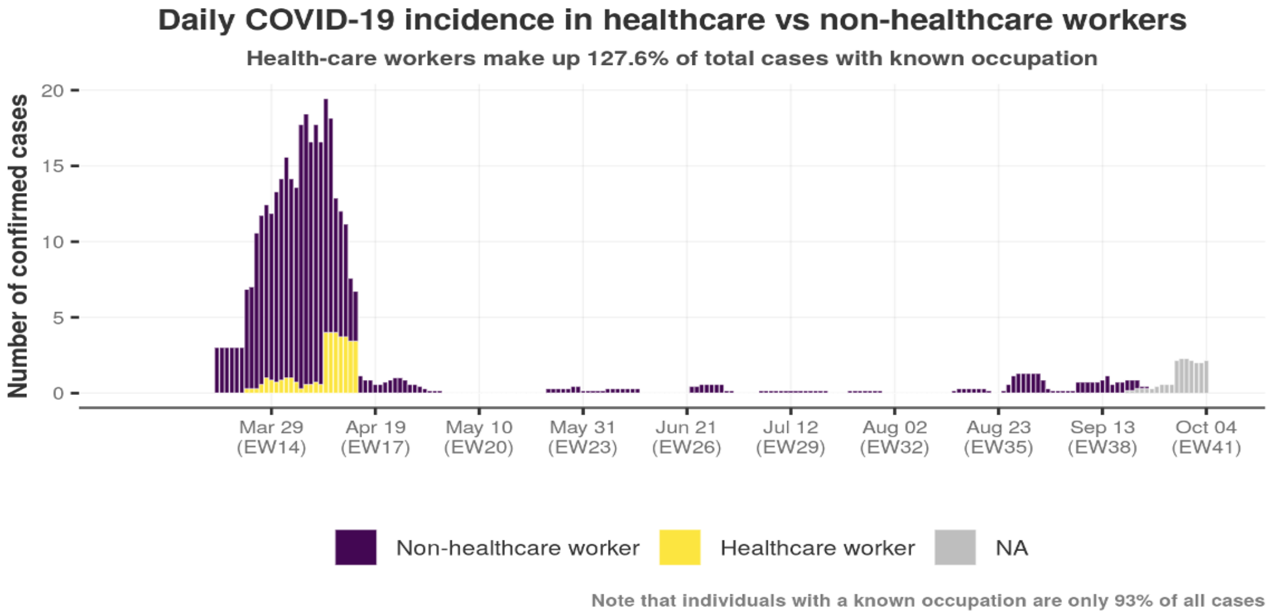

3. PHSMs

(Data updated 4 October 2020)

3. PHSMs

3.1 Further public health and social measures

The early management of the virus was initiated with a lockdown on 20 March, 2 days after the detection of the first case. The lockdown order was extended three times - on 30 March, 10 April and 1 May - before it was finally terminated on 31 May 2020[1]. In order to increase the population's compliance, the government maintained all wi-fi connections, doubled data availability and extended digital TV access at no extra cost[6]. In addition, they closed schools, parks and other attractions on 19 March[2]. On 20 March, the Cabinet of Ministers agreed to the price of hand sanitizers, respirators N95 and PPF2 type and masks being controlled under the Consumer Protection Regulations. The football league was terminated on 6 April[16].

The enhanced communication plan carried out by the Communication Committee played an important role in the containment of the outbreak. The daily communication with citizens worked as a reminder of the responsibility of individuals and helped to sensitize and engage the population. The authorities used several different methods of communication for non-pharmaceutical measures information, and this helped build trust with the public and supported the behavioural change, namely the use of surgical masks when in public and social and physical distancing[1]. To strengthen the sensitization of the population, the government collaborated with key stakeholders as community leaders, religious representatives, sociocultural associations, NGOs and civil societies[13]. The authorities also temporarily removed taxes on masks and hand sanitizers[17].

Furthermore, on 15 May 2020, the National Assembly passed the COVID-19 Bill and the Quarantine Bill in an attempt to protect public health and the sustainability of households, which specified the details of the transition process from the curfew by strengthening the surveillance control and health system preparedness. More specifically, the COVID-19 Bill aimed to shield the economy throughout the pandemic and prepare for the recovery post-COVID. The Quarantine Bill intends to avoid a new viral outbreak and support the prepardness and response of the country for a future pandemic[18]. This allowed the progressive reopening of economic and other activities with strict sanitary rules and added measures to avoid a resurgence of the disease.

Another important measure was the early support of the healthcare system. Apart from the use of dedicated quarantine facilities and the implementation of an organized surveillance system, the government allocated 5.3 million dollars to the Ministry of Health and Wellness in order to purchase new equipment and medical PPEs[10]. In

17. Goal, Mauritius terminates football season, 2020. Retrieved from https://www.goal.com/en/news/coronavirus-mauritius-terminates-football-season/1wz0obtlbt7js1uwi2y7xjd43x

18. Republic of Mauritius, COVID-19 Communiqués 2020. Retrieved from http://www.govmu.org/English/Pages/ViewAllCommuniquecovid19.aspx

19. Government Information Service, Prime Minister's Office (May, 2020) Covid-19 Bill and Quarantine Bill adopted at National Assembly Retrieved from http://www.govmu.org/English/News/Pages/COVID-19-(Miscellaneous-Provisions)-Bill-andQuarantine-Bill-adopted-at-National-Assembly.aspx

20. Republic of Mauritius (2018, April 09), Access to key health care services is every citizen's fundamental right, says Minister Husnoo. Retrieved from http://www.govmu.org/English/News/Pages/Access-to-key-health-care-services-is-every-citizen%E2%80%99s-fundamental-right,-says-Minister-Husnoo.aspx

21. Ministry of Social Integration, Social Security and National Solidarity. Social Aid and Unemployment Hardship Relief. (Accessed at 24 June 2020) Retrieved from http://socialsecurity.govmu.org/English/ServicesMenu/Pages/Social-Aid--Unemployment-Hardhip-relief.aspx

22. Mauritius chamber of Commerce and Industry, Business updates, 24 June 2020. Retrieved from https://www.mcci.org/en/media-news-events/business-updates/government-wage-assistance-and-self-employed-assistance-scheme/

23. UNDP (2020), Support to the National Response to Contain the Impact of COVID-19. Retrieved by https://www.undp.org/content/dam/rba/docs/COVID-19-CO-Response/Government_COVID_Responses_Mauritius_Seychelles_29March2020RB-2.pdf

24. Republic of Mauritius (2020), COVID-19 Communiqués, Retrieved from http://www.govmu.org/English/Pages/ViewAllCommuniquecovid19.aspx

addition, on 6 March, the authorities increased the testing capacity of the country and addressed issues regarding the availability of essential supplies. Training programs were initiated to aim the protection of healthcare workers[5].

3.2 Adherence to measures

The citizens of Mauritius have free access to healthcare services[19]. Furthermore, they have access to social aid and unemployment hardship relief offered by the Ministry of Social Integration, Social Security and National Solidarity[20]. The government also implemented a series of additional measures to protect more vulnerable groups. As a result, the Wage Assistance Scheme and Self-Employed Assistance Scheme were formed to support the population[21]. They ensured the distribution of food products to families in the Social Register[22]. Furthermore, on 25 March 2020, the Finance and Audit Act established the COVID-19 Solidarity Fund, which provides aid to any person, institution and program, or project affected by the outbreak[23]. As the measures were extended, the authorities maintained financial assistance to the citizens directly affected by the restrictions[3].

One of the reasons Mauritius was successful in containing the epidemic was, apart from the implementation of strict isolation measures and the deployment of a well-planned communication strategy, the focus on meeting the needs of the population. It is possible that this strategy ensured the adherence of the population to the measures.

4. Issues Affecting the Response

(Data updated 4 October)

4. Issues Affecting the Response

4.1 Socio-political And Economic challenges

4.1.1 Economic challenges

Mauritius' early preparedness and prompt response succeeded in containing the outbreak of COVID-19. However, the government has been facing several important challenges for the economy and wellbeing of citizens. As tourism is one of the main industries of the country, prolonged disruption could be harmful for the economy[26].

The authorities organised a comprehensive surveillance plan that enabled the tourism industry to resume activity in a safe way in the beginning of October. To this end, the ministry of tourism has published a series of sanitary measures and restrictions to be applied in the sector upon restarting activities[14]. Although, as the second phase of the border re-opening includes obligatory paid 14-day quarantine before allowing people to move freely around the island, it is not expected to attract the usual number of tourists. In addition, an extended operation like this one might prove difficult to manage in the long term, due to the personnel and resources needed to operate. Also, a central part of the plan is the cooperation of the private sector, more specifically tourism establishments, shipping and airports employees, and transportation providers[14].

4.1.2 Socio-political challenges

Considering that Mauritius has the highest rate of non-communicable diseases (NCDs)[5] in Africa and a high percentage of people older than 60 years of age (15% of the population)[4], a second wave of the disease could have serious implications on the health of the population. These parameters need to be considered when planning for restarting the tourism activities.

4.1.3 Food Security

Another important issue is food security. The country is highly dependent on food imports to cover local needs, and any issues that jeopardise or disrupt international trade could result in a food crisis[25]. The outbreak itself, and the series of regulations that were imposed, resulted in higher than normal daily costs that need to be addressed in the short and long term based on a clear strategy.

4.1.4 Gender-based Violence

There was an upsurge in domestic violence during the lockdown period with 520 cases reported from 20 March to 30 May 2020. Women accounted for 481 cases (92.5%) compared to 39 cases (7.5%) for men. By nature of problem physical assault recorded the highest share (40%) and followed by verbal abuse (33.2%). It is reported that 111 victims left the conjugal roof during the lockdown and all of them were women.

25. Ministry of Tourism (Accessed at 10 August 2020). Overview of Tourism sector in Mauritius. Retrieved from http://tourism.govmu.org/English/Pages/Overview-of-Tourism-Sector-in-Mauritius.aspx
26. International Trade Center, Mauritius National Export Strategy 2017-2021, (Accessed at 07 July 2020) http://industry.govmu.org/English/Documents/2_Agro-Processing_web.pdf

5. Interpretation &Recommendations

(Data updated 4 October 2020)

5. Interpretation & Recommendations

5.1 Situation Summary

The first three cases of COVID-19 in Mauritius were reported on 18 March. Since then, 395 cases and 10 deaths have been reported.

Initially, following several imported cases, local transmission was evident and predominated until April 2020. Through a well-structured response plan, Mauritius succeeded in containing local transmission, and avoided overwhelming the healthcare facilities as the number of patients requiring hospitalization or intensive care was kept under control. The early deployment of preventative and control measures played a key role to this end.

Currently, Mauritius is experiencing another challenge: imported cases arriving from repatriation services.
Since Mauritius' main economic activity is the tourism industry, borders may soon reopen to increase the economic growth, thereby also exposing the country more imported cases of COVID-19. The challenge for the country is to deal with imported cases, in order to maintain control over local transmission, while also keeping the economy stable.

Although the main international hub of Mauritius is Port Louis, most of the locally-acquired cases were observed in Plaine Wilhems, the most populated city, and most imported cases have occurred in Flacq district. However, there is no information on the origin of most cases imported in the last month.

Men have been overwhelmingly more at risk of both infection and death, and most cases have occurred in working-age classes. Interestingly, fourteen days after the increase of the disease incidence, a peak of COVID-19 was observed among HCWs. More precisely, 36 confirmed cases among HCWs from 22 March to 9 April. Since 10 April no infection among HCWs has been confirmed.

5.2 Short-Term Action

- Focus on the organization and preparation of the recommencement of the tourism industry by following the country guidelines.
- Continue to evaluate the effectiveness of the current measures and anticipate the needs after the complete restart of tourism activities to cover potential gaps.
- Evaluate the efficacy of screening mechanisms in the ports and airports and for contact tracing procedures. Also, evaluate new diagnosis tools to use in the borders such as rapid molecular tests.
- Improve and maintain contact tracing of sporadic cases ; if possible, share or publish data on contact tracing methods.
- Maintain preparedness for a possible second wave. Identify the gaps in available healthcare personnel, equipment and infrastructure to make informed decisions in the allocation of resources.
- Maintain good communication with the public.
- Continue to invest in the protection and training of healthcare workers.
- Evaluate the impact of COVID-19/lockdown on domestic violence, psychological status of the population, the

effect and status of NCDs, and ensure access of the population to necessary services.

- In case of hospitalized patients, it may also be recommended to collect more detailed clinical data on these patients in the future; or if no detailed data can be collected, record the number of newly hospitalized patients per day.
- Collect and publish individual level data on number of tests performed, test positivity rate, and potential delay in test turnaround time.
- Discuss experiences regarding the measures adopted by Mauritius with other African countries, mostly with the other regional insular countries, with demographic and economic similarities, to improve the COVID-19 pandemic control in this region.

5.3 Long-Term Action

- Design a clear long-term strategy for the absorption of unexpected costs incurred during the pandemic.
- Identify the vulnerable population groups and those most impacted by the pandemic, and develop a long-term plan to support them.
- Evaluate the social impact of the outbreak and identify ways to mitigate the results.
- Invest in strengthening food production in the country and decreasing dependency on importation of goods.
- Plan strategies for the alternate use of the new laboratorial and hospital infrastructures acquired during the pandemic.
- Plan surveillance strategies for new epidemics, considering the knowledge and infrastructure acquired during the current pandemic.
- Evaluate data consortia with other African countries and/or other regions to study the COVID-19 pandemic
- Evaluate catch-up immunization program (as well as other health programs), considering the possible gap that occurred during the lockdown.

6.Appendix

(Data updated 4 October 2020)

6. Appendix

Overview of case counts, death counts and CFR for all WHO AFRO countries (Data Updated 4 October 2020)

Country	Cases	Deaths	Crude CFR	Population	Cases per million	Deaths per million
South Africa	681289	16976	2.5	56697608	12016	299.4
Ethiopia	78819	1222	1.6	112833693	698.5	10.8
Nigeria	59345	1113	1.9	207185471	286.4	5.4
Algeria	52136	1760	3.4	43012658	1212	40.9
Ghana	46829	303	0.6	30536428	1534	9.9
Kenya	39427	731	1.9	52314849	753.6	14
Cameroon	20875	418	2	27884774	748.6	15
Cote d'Ivoire	19849	120	0.6	25599373	775.4	4.7
Madagascar	16558	232	1.4	27797000	595.7	8.3
Senegal	15094	312	2.1	17452400	864.9	17.9
Zambia	15052	333	2.2	18918307	795.6	17.6
Namibia	11626	123	1.1	2743745	4237	44.8
DRC	10760	274	2.5	94058574	114.4	2.9
Guinea	10754	66	0.6	14352102	749.3	4.6
Mozambique	9196	66	0.7	31995850	287.4	2.1
Uganda	8808	81	0.9	49051105	179.6	1.7
Gabon	8797	54	0.6	1915836	4592	28.2
Zimbabwe	7888	228	2.9	17449538	452	13.1
Mauritania	7520	161	2.1	4546376	1654	35.4
Cabo Verde	6360	65	1	552351	11514	117.7
Malawi	5786	179	3.1	19998214	289.3	9
Eswatini	5569	111	2	1359068	4098	81.7
Angola	5370	185	3.4	29240847	183.6	6.3
Congo (Rep)	5089	89	1.7	5950251	855.3	15
Eq. Guinea	5045	83	1.6	967760	5213	85.8
Rwanda	4866	29	0.6	13117385	371	2.2
CAF	4845	62	1.3	20735261	233.7	3
Gambia	3594	115	3.2	2335718	1539	49.2
Mali	3184	131	4.1	20525117	155.1	6.4
Botswana	3172	16	0.5	2449622	1295	6.5
South Sudan	2726	50	1.8	14839454	183.7	3.4
Guinea-Bissau	2362	39	1.7	2118558	1115	18.4
Benin	2357	41	1.7	12488112	188.7	3.3
Sierra Leone	2269	72	3.2	7119979	318.7	10.1
Burkina Faso	2154	59	2.7	20653498	104.3	2.9
Togo	1854	48	2.6	8262867	224.4	5.8
Lesotho	1639	38	2.3	2251094	728.1	16.9
Liberia	1348	82	6.1	5095339	264.6	16.1
Chad	1217	86	7.1	22545704	54	3.8
Niger	1200	69	5.8	36399415	33	1.9
Sao Tome e P	913	15	1.6	210613	4335	71.2
Burundi	514	1	0.2	13179830	39	0.1
Tanzania	509	21	4.1	64577992	7.9	0.3
Comoros	487	7	1.4	883162	551.4	7.9
Eritrea	398	0	0	5930313	67.1	0
**Mauritius**	**395**	**10**	**2.6**	**1291483**	**298.1**	**7.7**
Seychelles	142	0	0	98885	1436	0

Overview of case counts and death counts across WHO AFRO regions

Region	Cases	Cases per million	Deaths	Deaths per million
Southern Africa	746587	4077	18255	99.7
Western Africa	186073	448.1	2796	6.734
**Eastern Africa**	**152648**	**446.5**	**2376**	**6.95**
Central Africa	58055	309.7	1082	5.772
Northern Africa	52136	1212	1760	40.92

Plot of growth rate each week

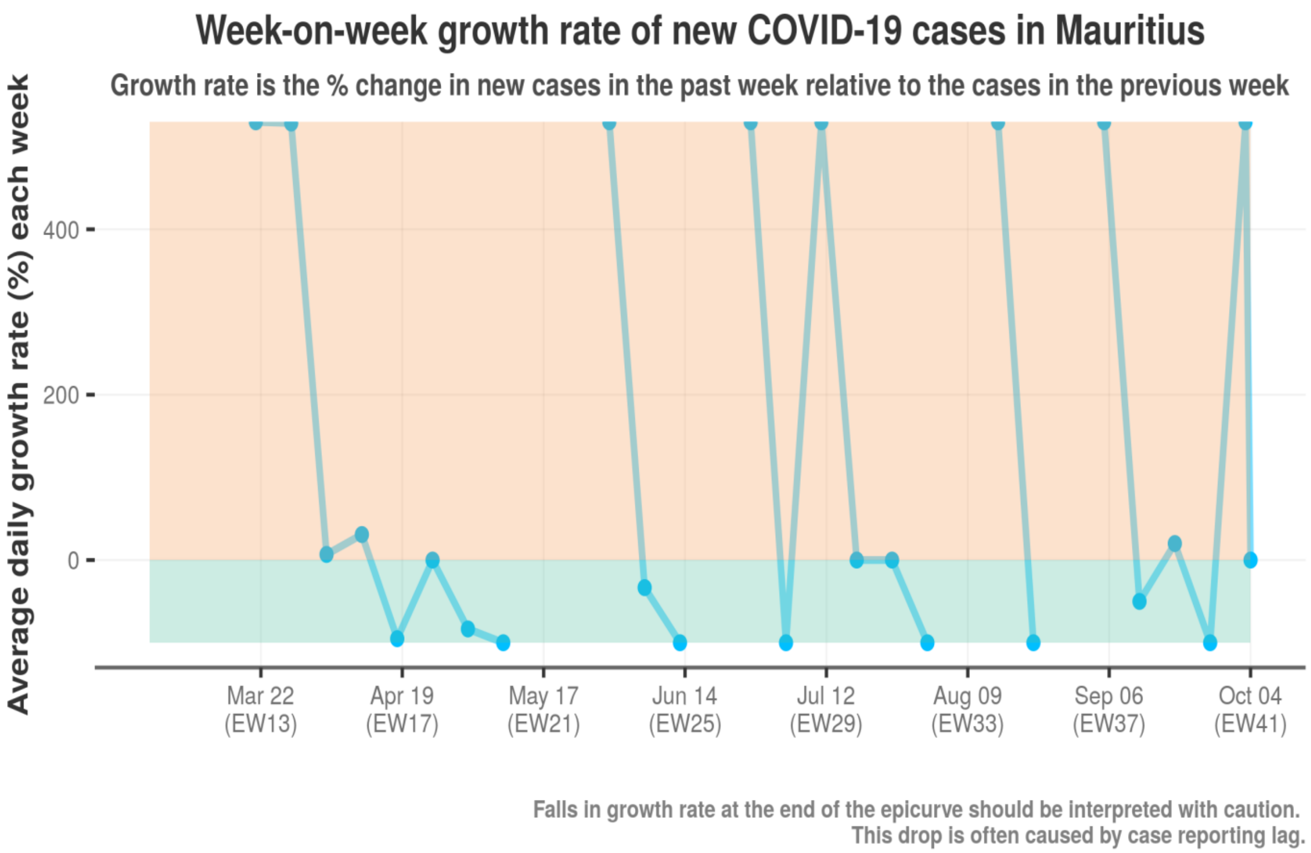

Epidemic Progression Maps for Mauritius (Imported versus Locally-acquired)

Imported Cases

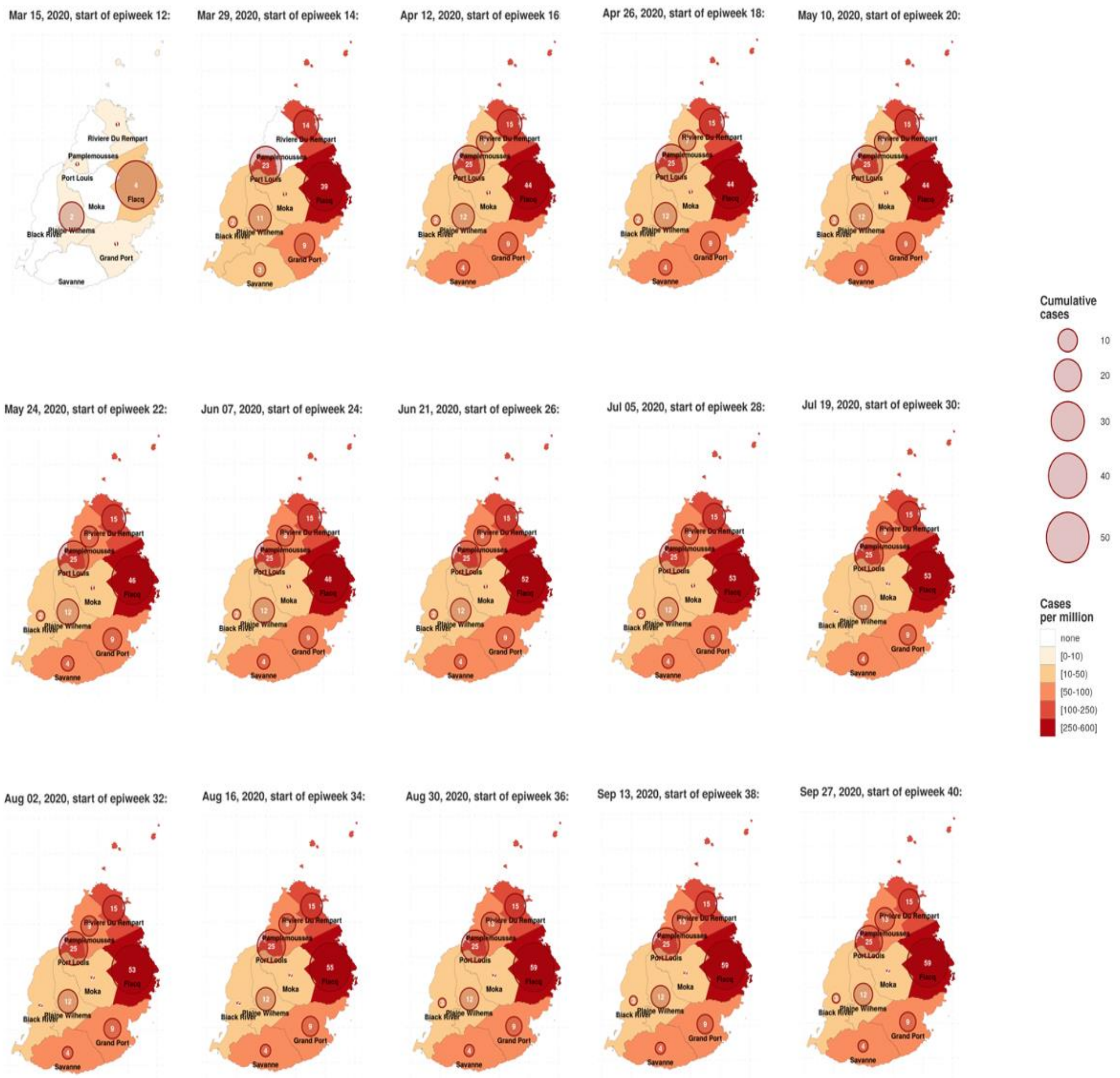

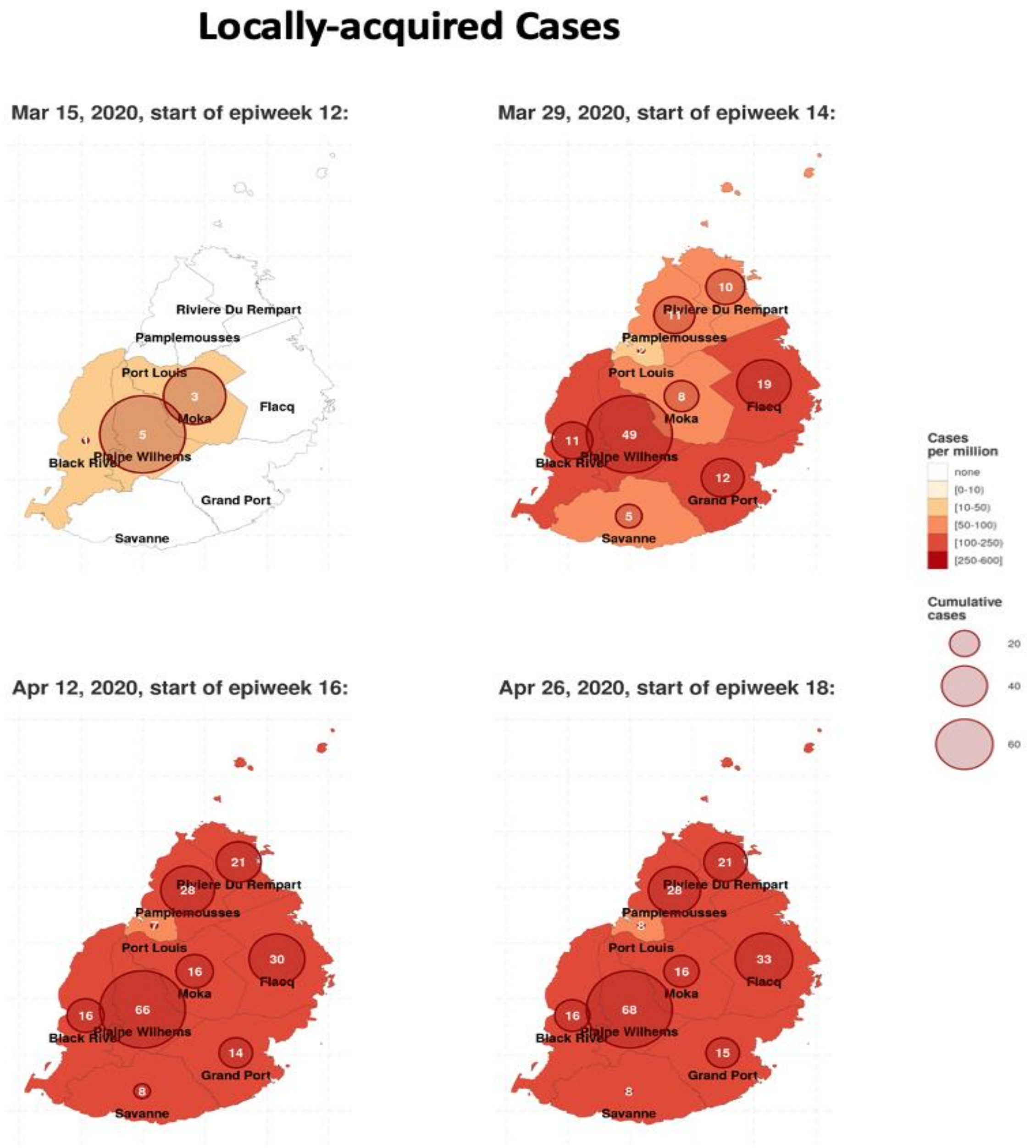

World Health Organization

Regional Case Summary

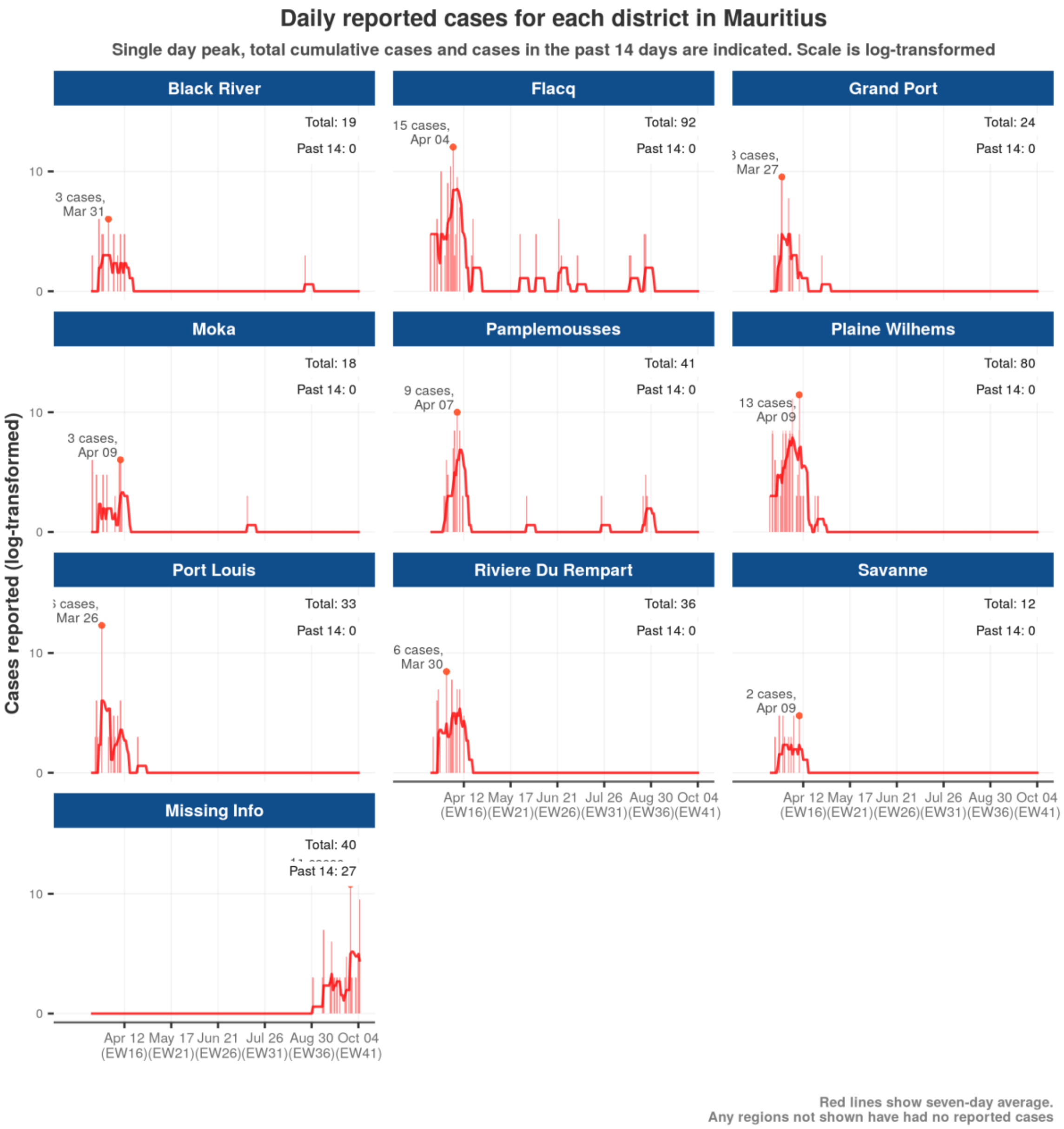

World Health Organization

Regional Death Summary

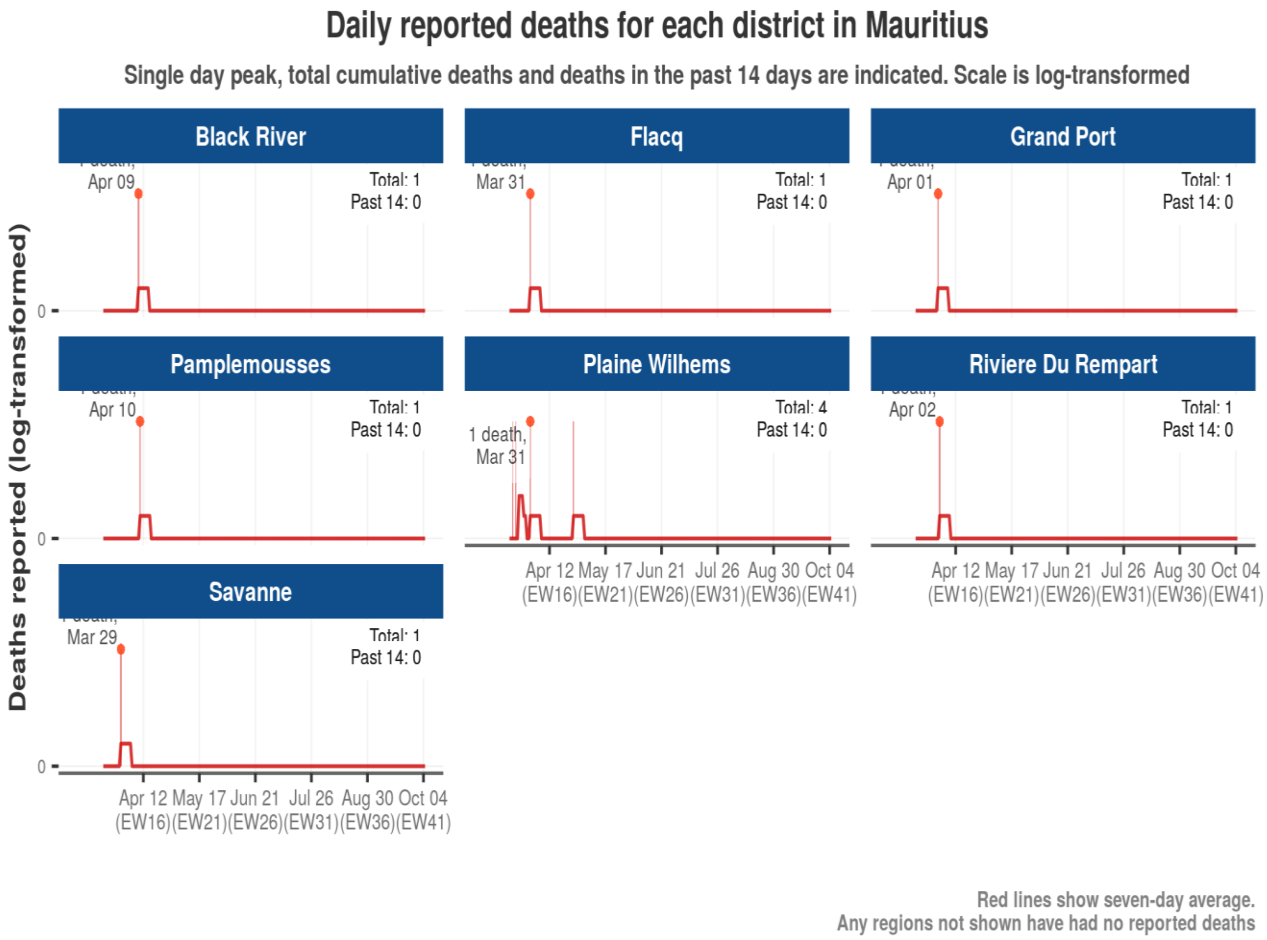

Acknowledgements

Project Management and Coordination:
Olivia Keiser, Sara Botero Mesa.

Data preparation (in country): Ministry of Health and Wellness, Mauritius :
Director Health Services (Public Health):
Dr V. Gujadhur and Dr V.Dinassing (Directr Health Services), Dr F.M. Khodabocus (RPHS), Dr R.P. Ramhith (RPHS), Mr M. Teeluck (Epidemiologist), Dr L.P. Veerapa-Mangroo (Community Physician and Field Epidemiologist), Dr Z. Suffee (Community Physician), Dr R. Luchmiah (MHO+MPH), Mr A. K.S. Seeburn (Statistician), Mrs M. Meerun (Health Surveillance Officer), Mrs Z. Johar (Health Surveillance Officer)

Data cleaning and Analysis: Jessica Lee Abbate, Flavio Coelho, Lucas Bianchi, Daniel Camara, Izabel Reis, Kene Nwosu, Erol Orel, Maroussia Roelens, Adrian Lison, Beni Broohm, Adit Kaneria,.

WHO regional office Africa: Benido Impouma, Franck Mboussou, Charles Okot, William George, Opeayo Ogundiran.
WHO Country Office, Mauritius : Dr L. Musango (WHO Representative), Mr A. Nundoochan (Incident Manager), Mrs V. Vythelingum(Health Promotion and NCDs Officer) and Dr F. Shaikh (Technical Officer HSS).

Writing and Edition: Timokleia Kousi, Cristina Barroso Hofer
First draft of report: Timokleia Kousi
Editing: Azizah Siddiqui, Rubi Scalit
Review: Cristina Barroso Hofer, Olivia Keiser

This report benefited from valuable inputs and support from many colleagues. Particular thanks go to:, Elise Blandenier, Stéphane Marchand-Maillet, Verena Carrara, Morteza Mahdiani, Camille Beatrice Valera, Cyril Pervilhac, Gabriela Guizzo Dri, Plamenna Venkova, Paola Anabalon, Julius Ssempiira, Yai-Ellen Gaye, Artur Muloliwa, Bertil Wicht, Emile Haas, Michael Adhanom, Daniela Pires Ferreira Vivacqua, Leckson Mukavhi, Wingston Ng'ambi Christine Choirat, Olivier Verscheure, Beat Stoll, Manuel Wiederkehr, Kayleigh Gallagher, Cleophas Chimbetete, Hemanth Reddy, Olena Wagner, Paula Anabalon, Zahra Habibi, Isotta Triulzi, Papy Ansobi, Artur Muloliwa, Papa Amadou, Benedict Nguimbis, Martin Alexis, Lou Locci, Kathleen Lindsey, Sabina Rodriguez, Akarsh Venkatasubramanian, Health Solutions Research (HSR), Association Actions en Santé Publique (AASP), University of Geneva, Institute of Global Health.

The analyses were run on RenkuLab (http://renkulab.io/), a free and open platform for collaborative and reproducible data-driven science developed by the Swiss Data Science Center (https://datascience.ch/).
