# Supplementary material for: Leveraging human resources for outbreak analysis: lessons from an international collaboration to support the sub-Saharan African COVID-19 response
Source: BMC Public Health. 2022 May 31;22:1073. doi: 10.1186/s12889-022-13327-1 (PMC9152815; doi:10.1186/s12889-022-13327-1)
Supplement: Supplementary file 2 — Additional file 2. Web survey. Survey answered by postgraduate students, in-country experts, and other global health professionals. [file 12889_2022_13327_MOESM2_ESM.pdf]

Expert’s survey

1. How would you describe your experience collaborating with the GRAPH network?
2. Please describe 2 positive experiences you had and 2 aspects that can be improved about the WHO/GRAPH network (ASP) collaboration
3. Would you recommend the GRAPH network to support epidemiological responses in different contexts or organizations. and why?
4. Addressed to African Experts. Would you recommend the GRAPH Network to support the epidemiological response in your country (with the Ministry of Health), and why?
5. Country of residency (optional)

Survey Reporters and Global Health students

1. How would you describe your experience collaborating with the GRAPH network?
2. What aspect of your experience with the GRAPH network do you find the most interesting?
3. How your experience in the GRAPH network contribute to your Global Health training?
4. Please describe 2 positive experiences you had and 2 aspects that can be improved about the WHO/GRAPH network (ASP) collaboration
5. Would you recommend the GRAPH network to support epidemiological responses in different contexts or organizations, and why?

Data Analysts Survey

1. Data analysis expertise: Senior or Junior
2. How would you describe your experience collaborating with the GRAPH network?
3. What aspect of your experience with the GRAPH network do you find the most interesting?
4. For Junior Analysts. Did this experience have an impact in your data analysis skills? if yes could you explain how?
5. Please describe 2 positive experiences you had and 2 aspects that can be improved about the WHO/GRAPH network (ASP) collaboration
6. Would you recommend the GRAPH network to support epidemiological responses in different contexts or organizations, and why?

Africains_analystes de données_Survey_(Ttraining program)

1. Comment décririez-vous votre expérience en tant que collaborateur du GRAPH Network ?
2. Avez-vous trouvé notre programme de formation utile? Pourquoi?
3. Cette expérience a-t-elle eu un impact sur vos compétences en matière d'analyse de données, et comment ?
4. Quel aspect de votre expérience avec le réseau GRAPH trouvez-vous le plus intéressant ?
5. Veuillez décrire 2 expériences positives que vous avez eues et 2 aspects qui peuvent être améliorés concernant la collaboration entre le GRAPH Network et l'OMS (ASP)
6. Recommanderiez-vous GRAPH Network pour soutenir la réponse épidémiologique dans votre pays (avec le ministère de la santé), et pourquoi ?
