# Supplementary material for: Leveraging human resources for outbreak analysis: lessons from an international collaboration to support the sub-Saharan African COVID-19 response
Source: BMC Public Health. 2022 May 31;22:1073. doi: 10.1186/s12889-022-13327-1 (PMC9152815; doi:10.1186/s12889-022-13327-1)

A PROPOSAL BY

GRAPH NETWORK

PROJECT

WEB APPLICATION TO ADVANCE GLOBAL EPIDEMIC RESPONSE.

PROJECT PROPOSAL

CONTACT

PROFESSOR OLIVIA KEISER PHD.
INSTITUTE OF GLOBAL HEALTH,UNIVERSITY OF GENEVA
PROJECT DIRECTOR @THEGRAPHNETWORK
PHONE: +41 22 379 41 79
EMAIL: OLIVIA.KEISER@UNIGE.CH

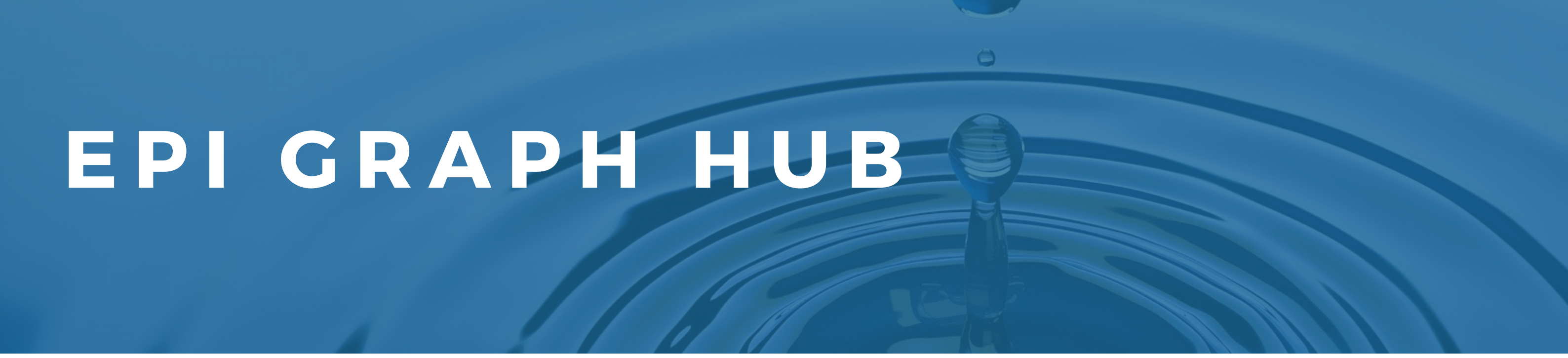

EPI GRAPH HUB

PROJECT DESCRITPION

The Global Research and Analyses for Public Health (GRAPH) network aims to build a sustainable, multidisciplinary collaborative network to support disease surveillance and data analysis for public health risks across the globe. Training of local researchers and reinforcing strong links to country governments and the World Health Organization (WHO) are central pillars of the network's activity.

Since 1 June 2020, our team has been engaged in a WHO-funded effort to track the progression of COVID-19 in Africa and provide actionable insights supporting pandemic response efforts. This involves the cleaning and analysis of various health data - including case counts, testing data, partial patient history - provided through the WHO Africa office and from the Ministries of Health of member states (32 and counting) who have asked the regional office for assistance. During this period we have produced 34 highly detailed epidemiological reports which were shared with the countries.

The emergence of SARS-CoV-2 found the world both in need of, and under-prepared for, the implementation of rapid response tools for coordinating surveillance and mitigation efforts. Obstacles to the flow of information has very likely meant unnecessary loss of both lives and economic stability. We have been working to remove these obstacles and develop tools that are both informative and easy to implement in crisis situations. Our work will have implications for worldwide disease prevention, surveillance, and rapid mitigation of new epidemics.

THE OBSTACLES

Even with good communication between institutions, extracting knowledge from raw data requires a substantial amount of manipulation as it often follows no standard format across countries, sometimes varying even within countries from one submission to the next. Common problems encountered range from the large variety of data formats used for dates to differences in naming conventions for administrative regions, to typos or misplacement of information that can happen when filling the data across columns. Language is also a key challenge as variable names, such as for medical conditions, are different in different languages, and require standardization.

Although a standardized data collection (spreadsheet) template was provided by WHO-Afro, a lot of time is still required to clean and verify the quality of the data. The lack of validation mechanisms at the data entry stage, means that a post-hoc data cleaning and validation is indispensable. Manual cleaning this data is time-consuming and error-prone. Our data-science team has dedicated many hours to develop an effective automated process, working on a country-by-country basis.

This need for manual data cleaning and verification delays the availability of decisions in crisis situations where the information should be updated on a daily - or preferably real-time - basis. These discrepancies and inconsistencies are common in all projects that involve data from various sources. However, during a pandemic, the time spent has a higher cost than at any other moment. The shorter this process of cleaning can be, the more people could be protected in critical situations, as decision makers can rely on qualitative real time data to guide their policies.

EPI GRAPH HUB

OUR VISION

We build strong relationships with people on the ground who are key to facilitating the exchange of information, provide essential contextual insights, and most importantly, support the analysis itself by applying their expertise. These partners are mainly people working in a health related institution or in the statistics field, and they do not simply participate but also take on leadership roles in the network. These in-country partners are crucial for helping to identify organisational problems that block the process of information exchange and work towards negotiating solutions.

During the pandemic we saw the rise of many health dashboards that were reporting the covid cases in various places in the world. Many of these tools are only visualization of scraped data or open data that were sometimes compiled by a unique developer, showing the power of clean open data available for projects.

We go a significant step further in the development of such projects, by working on the whole pipeline, from data collection to rich, meaningful and actionable analysis. More than just another application or dashboard, we are a coordinated grassroots engine for the integration and communication of indicators identified by local researchers and decision-makers. By providing flexible, user-friendly, and automated tools, the time span between the data collection and report generation, where actionable insights are returned to decision-makers and on-the-ground clinicians, can be greatly reduced.

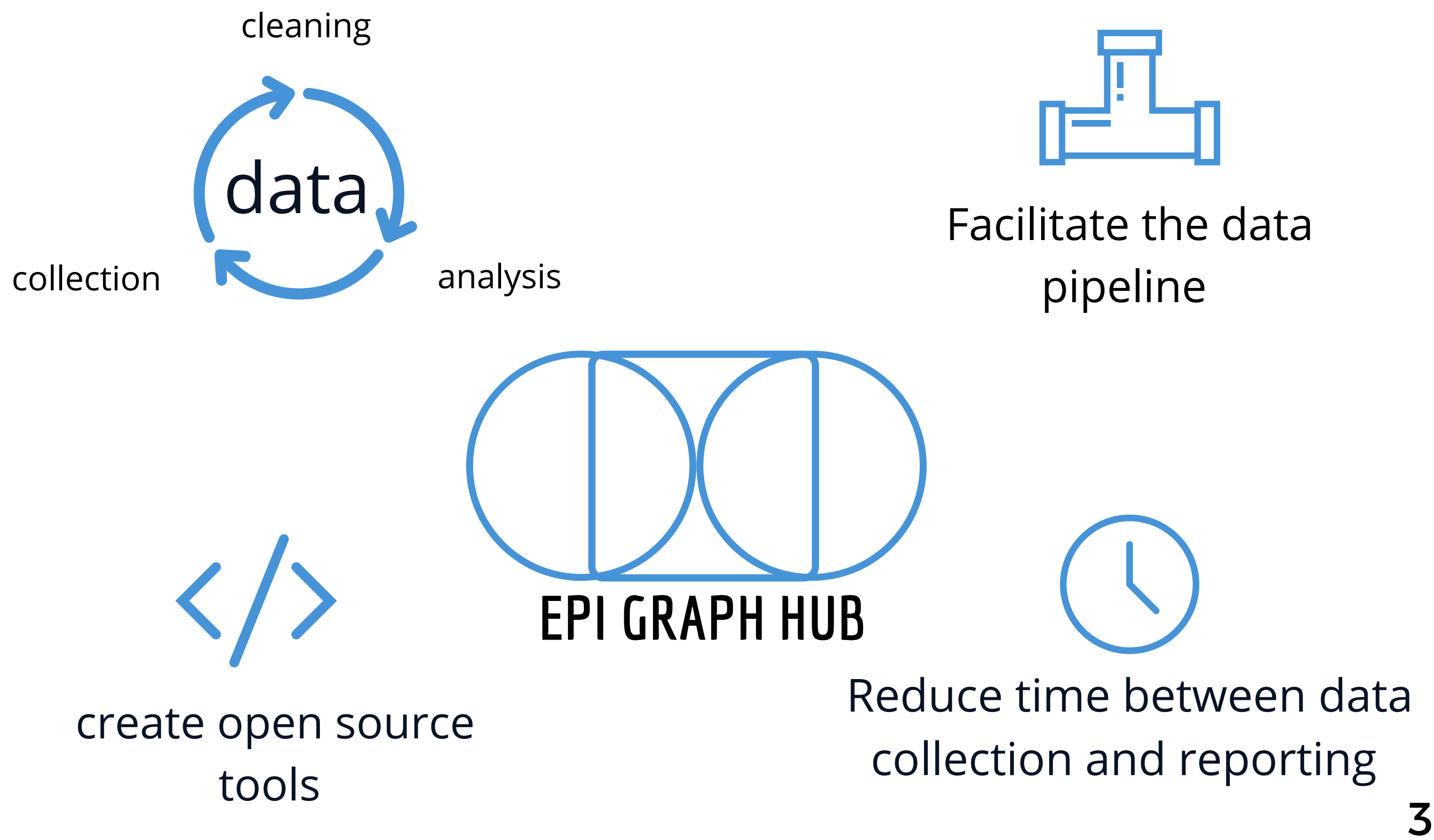

EPI GRAPH HUB

OUR SOLUTION

Based on our observations during COVID-19 response and prior work, we've developed an open source web application (the "Epi-GRAPH Hub", including a web-portal and integrated mobile app) using Shiny and R that combines the processes of aggregating data from multiple sources, cleaning, visualizing, analyzing and reporting. More than a simple dashboard, this application automates the generation of customized reports based on all the possible combinations of the information that was extracted from the data. In addition, the application is capable of identifying the key indicators needed by public health decision-makers and on-the-ground responders rapidly during a crisis.

We will integrate our project with the public health infrastructures already in place, namely the data-collection and management systems of each country. Most countries in Africa have operational information systems for data collection. We will develop software to connect with these health information systems, automate the operation of our pipeline of cleaning, visualization, and generation of epidemiological reports for outbreak response. This integration will further help optimize the data collection and report generation process.

The Epi-GRAPH Hub Solution is a living solution that can evolve in a manner responsive to the needs of individual countries, supported by our in-country partners, while maintaining the ability to provide consistent and actionable public health reports. The GRAPH Network was conceived from the start to work alongside national governments to improve their own analytical infrastructure and personnel. Once this integration is complete, governments will be able to appropriate the initiative and use it independently. Another goal of our network is the strengthening of local capacity for epidemiological data analysis. For that we have developed an in depth training module to be deployed in parallel with our data analysis pipeline.

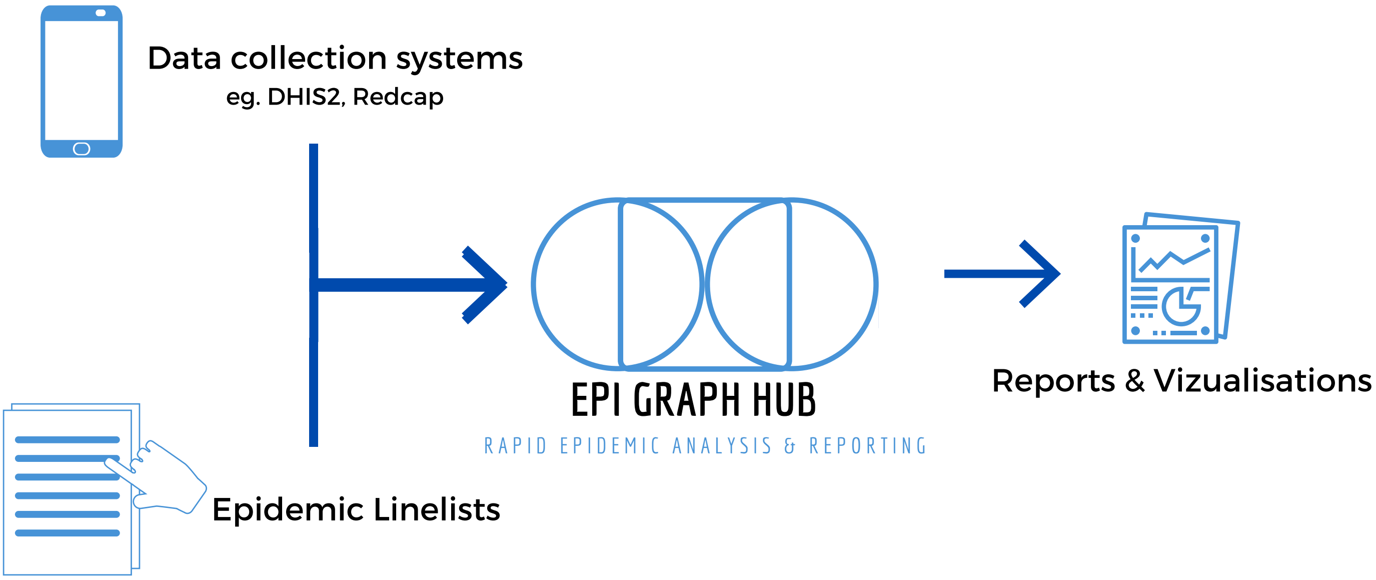

Supplement: Supplementary file 3 — Additional file 3. EpiGRAPH Hub Concept sheet. [file 12889_2022_13327_MOESM3_ESM.pdf]
